# The role of agrin as a downstream mediator of Slit3 in osteogenic-angiogenic interaction under osteoporosis

**DOI:** 10.1093/rb/rbag192

**Published:** 2026-09-09

**Authors:** Xu Ye, Zhenghao Xu, Gan Zhong, Wenge He, Yichi Zhang, Leilei Qin, Cheng Chen, JianYe Yang, Li Chen, Huan Yao, Yingkun Hu, Chengqiang Zhou, Qinyang Zhang, Huimei Liang, Xuan Gong, Denghui Xie, Baicheng He, Zhong Alan Li, Ning Hu

**Affiliations:** Department of Orthopedics, The First Affiliated Hospital of Chongqing Medical University, Chongqing 400016, China; Department of Biomedical Engineering, The Chinese University of Hong Kong, Hong Kong SAR 999077, China; Department of Orthopedics, The First Affiliated Hospital of Chongqing Medical University, Chongqing 400016, China; Department of Orthopedics, The First Affiliated Hospital of Chongqing Medical University, Chongqing 400016, China; Department of Orthopedics, The First Affiliated Hospital of Chongqing Medical University, Chongqing 400016, China; Department of Orthopedics, The First Affiliated Hospital of Chongqing Medical University, Chongqing 400016, China; Department of Biomedical Engineering, The Chinese University of Hong Kong, Hong Kong SAR 999077, China; Department of Orthopedics, The First Affiliated Hospital of Chongqing Medical University, Chongqing 400016, China; Department of Orthopedics, The First Affiliated Hospital of Chongqing Medical University, Chongqing 400016, China; Department of Orthopedics, The First Affiliated Hospital of Chongqing Medical University, Chongqing 400016, China; Orthopedics Department, Chongqing University Fuling Hospital, Chongqing 408107, China; Department of Orthopedics, The First Affiliated Hospital of Chongqing Medical University, Chongqing 400016, China; Department of Orthopedics, The First Affiliated Hospital of Chongqing Medical University, Chongqing 400016, China; Department of Orthopedics, The First Affiliated Hospital of Chongqing Medical University, Chongqing 400016, China; Department of Orthopedics, The First Affiliated Hospital of Chongqing Medical University, Chongqing 400016, China; Department of Orthopedics, The First Affiliated Hospital of Chongqing Medical University, Chongqing 400016, China; Department of Orthopedics, The First Affiliated Hospital of Chongqing Medical University, Chongqing 400016, China; Department of Outpatient Care, Chongqing General Hospital, Chongqing 401147, China; Department of Joint Surgery and Sports Medicine, Center for Orthopedic Surgery, The Third Affiliated Hospital of Southern Medical University, Orthopedic Hospital of Guangdong Province, Guangzhou 510630, China; Department of Pharmacology, Chongqing Medical University, Chongqing 400016, China; Department of Biomedical Engineering, The Chinese University of Hong Kong, Hong Kong SAR 999077, China; Department of Orthopedics, The First Affiliated Hospital of Chongqing Medical University, Chongqing 400016, China

**Keywords:** Slit3, Agrin, osteo-angiogenic interaction, organ-on-a-chip, osteoporosis

## Abstract

Osteoporosis, characterized by an imbalance between bone formation and bone resorption, is currently treated mainly by inhibiting resorption or promoting formation. However, these therapeutic strategies are often limited in efficacy because they fail to simultaneously target the coupled osteogenic and angiogenic processes in bone metabolism. In recent years, Slit3 has emerged as a key regulator of osteo-angiogenic interaction and is now considered a promising therapeutic target for metabolic bone disease. In the present study, we performed *in vitro* and *in vivo* experiments, including employing a human cell-derived microfluidic organ-on-a-chip (OoC) platform to investigate the upstream and downstream regulatory relationships within the Slit3–Agrin axis in osteo-angiogenic interaction, performing functional assays to elucidate the signaling pathway between Slit3 and Agrin, and establishing a pathological animal model to validate the osteogenic effect of this axis. The results showed that Slit3 mediates the nuclear translocation of β-catenin via the Robo1 receptor. Subsequently, β-catenin binds to TCF3 to activate the transcription of Agrin. Agrin then binds to LRP4 to negatively regulate the canonical Wnt/β-catenin signaling pathway, thereby orchestrating osteogenesis. This study reveals that the Slit3–Agrin axis serves as a core regulator of the bone-vascular unit and demonstrates the promising potential of OoC-based systems for developing targeted therapies.

## Graphical Abstract

**Graphical Abstract:**
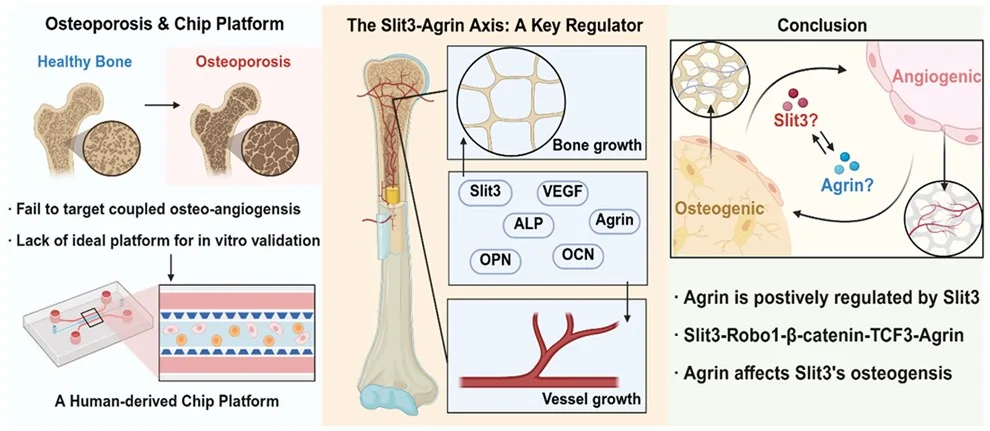

## Introduction

Osteoporosis is a metabolic bone disease characterized by reduced bone mass and degeneration of bone microstructure [1]. Globally, it has become a major public health challenge. The underlying pathological mechanism, an imbalance between bone formation and bone resorption, has been widely recognized. However, current mainstream clinical treatment strategies, such as inhibiting bone resorption by blocking RANKL, or promoting bone formation, are often limited in efficacy because they do not address the tightly coordinated processes of angiogenesis and osteogenesis in bone metabolism [2]. Consequently, the research focus has progressively shifted from individual cellular events to the complex bone microenvironment, in which the interplay between angiogenesis and osteogenesis constitutes a pivotal yet incompletely characterized process.

Slit3 has recently emerged as a pivotal regulator of osteo-vascular communication. Osteoblast-derived Slit3 promotes H-type vessel formation, enhances osteogenesis, reverses osteoporotic bone loss and accelerates fracture repair [3]. Since then, the central role of Slit3 in bone regeneration has been increasingly recognized. In 2023, Gao *et al*. [4] revealed that TPPU, a soluble epoxide hydrolase inhibitor, activates the Slit3/HIF-1α signaling pathway via the YAP1-ROBO1 axis, thereby promoting osteo-angiogenic coupling. More importantly, a study published in 2025 has already incorporated Slit3 as an interventional molecule into scaffolds to reduce fibrosis at the bone defect site and promote the formation of H-type vessels [5]. Collectively, these studies demonstrate that Slit3 acts as an indispensable molecular bridge linking the skeletal and vascular systems, and elucidating its regulatory mechanism holds profound significance for the development of more effective osteoporosis therapies. However, conventional research strategies remain predominantly restricted to the independent assessment of discrete cellular events, a reductionist paradigm that inherently precludes a comprehensive characterization of the bone microenvironment. The emergence of organ-on-a-chip (OoC) technology, also referred to as micro-physiological systems, provides a turning point to overcome this challenge. These systems integrate human cells, microfluidic technology and engineering biomaterials to construct *in vitro* models that feature 3D architecture, dynamic fluid perfusion and multi-tissue-level interactions, thereby effectively replicating the key physiological characteristics and complex microenvironments of human organs [6–8]. Such platforms not only effectively overcome the limitations of traditional 2D cell cultures and animal models in terms of species differences, physiological relevance and dynamic regulatory capability, but also allow precise control of individual experimental variables while preserving tissue structure and function.

This study first identified Agrin, a potential target positively regulated by Slit3, through integrated omics analysis. We then constructed an intermediary platform based on human-derived cells and OoC technologies to perform an initial validation of the Slit3–Agrin axis. This platform quickly enabled us to identify the precise regulatory relationships of the target under micro-physiological conditions, thereby facilitating subsequent validation. Following successful confirmation on the chip, we performed *in vitro* and *in vivo* experiments to explore the molecular mechanism and to confirm the findings. By integrating the human relevance of the OoC platform, the mechanistic resolution of conventional cell models, and the physiological complexity of animal models, this study establishes a cross-validated experimental framework for investigating osteo-angiogenic regulation (Figure 1). The aim of the present study was to evaluate the Slit3–Agrin axis from multiple aspects, including a human-derived platform, and then to verify its osteo-angiogenic interaction effects *in vitro* and *in vivo*, potentially providing new strategies for the development of osteoporosis therapies.

## Materials and methods

### Cell culture

Primary bone marrow mesenchymal stem cells (BMSCs) were isolated from the femurs and tibias of C57BL/6 mice, which were used to assess the osteogenic capacity of different molecules. Mouse lymph node endothelial cells (SVEC4-10, Procell system, #CL-0221) were commercially obtained to evaluate the angiogenic ability of different molecules. Human umbilical vein endothelial cells (HUVEC, FuHeng, #FH1122) and human mesenchymal stem cells (hMSCs, Lonza, #PT-2501), were commercially employed to construct the *in vitro* microfluidic chip platform for testing the Slit3–Agrin axis. For cell culture, all frozen cells were removed from a −80°C freezer and rapidly thawed in a 37°C water bath. The cell suspension was centrifuged at 1500 rpm for 3 min, and the supernatant was discarded. The cell pellet was resuspended in pre-warmed complete medium and seeded at a density of 5000 cells/cm^2^ in a T75 cell culture flask. hMSCs and BMSCs were cultured with Dulbecco's Modified Eagle Medium (DMEM) supplemented with 10% fetal bovine serum and 1% penicillin–streptomycin solution. HUVEC and SVEC4-10 cells were cultured with α-MEM supplemented with the same additives. All cells were maintained in a humidified incubator at 37°C with 5% CO_2_, and the culture medium was replaced every 48 h. All subsequent experiments used cells at passage 6.

### Transduction

Adenovirus (Ad), lentivirus (Lv) and recombinant adeno-associated virus (rAAV), all provided by OBiO, were used to mediate the overexpression of Slit3 in BMSCs, hMSCs and animal models. Taking LV-mediated transduction as an example, the procedure was performed as follows. Briefly, LV stored at −80°C was taken out and quickly placed in a 4°C ice-water bath for partial thawing. The virus was then diluted with pre-cooled complete medium at a volume ratio of 1:100. Polybrene-plus infection enhancer was diluted with the medium at a 1:10 ratio. The diluted virus, diluted Polybrene-plus and fresh pre-warmed complete medium were mixed at a volume ratio of 1:1:60. After gentle vortexing, the mixture was added to a well of a six-well plate and incubated in a humidified incubator at 37°C with 5% CO_2_ for 4–7 days to allow stable integration and transgene expression. Transduction efficiency was evaluated by fluorescence microscopy and by detecting Slit3 mRNA levels via RT-qPCR. Once successful transduction was confirmed, the cells were used for subsequent experiments.

### mRNA and secretory protein sequencing

To identify the role of Slit3 during osteogenesis, we performed mRNA sequencing on mouse BMSCs in the osteogenic inductive medium (OIM) group (Procell system, #PD-003) and the control group. To explore the downstream mediators of Slit3, we also conducted a combined analysis of transcriptomic and secretory proteomic data, comparing the OIM group and the OIM + Ad-Slit3 group. All data were subsequently analyzed using bioinformatics and statistical methods.

### Cell viability assay

Before constructing the chip platform, we first assessed cell viability. Cells were seeded at a density of 5 × 10³ cells/cm^2^ in a 6-well plate and cultured at 37°C with 5% CO_2_ for 24 h until they had fully adhered. The medium was discarded, and the cells were washed three times with PBS buffer. According to the instructions of the Calcein-AM/PI double staining kit (Solarbio, #CA1630), Calcein-AM and PI were diluted in PBS buffer at a volume ratio of 2:3:1000 to prepare the working solution. About 1 mL of the working solution was added to each well, and the cells were incubated at 37°C in the dark for 45 min. Images were immediately acquired using a Leica Stellaris 8 laser scanning confocal microscope with excitation at 488 and 561 nm. Six non-overlapping fields of view were randomly selected from each of three independent samples, and the fluorescent signals were automatically identified and counted using the Analyze Particles function in ImageJ software.

### CCK-8 assay

To evaluate the metabolic activity of cells and to determine whether direct co-culture of HUVECs and hMSCs elicits toxic effects through cell–cell interactions, a CCK-8 assay was performed at designated time points. HUVECs, hMSCs and Lv-Slit3-hMSCs were separately digested, collected and resuspended. For the experimental group, HUVECs were mixed with Lv-Slit3-hMSCs at a ratio of 1:1 in DMEM. For the control group, HUVECs were mixed with hMSCs at the same ratio. The cell suspensions were seeded into a 96-well plate at a density of 5 × 10^3^ cells/100 μL per well with four biological replicates per group. The blank group contained only culture medium and CCK-8 reagent. After overnight incubation at 37°C in a humidified atmosphere of 5% CO_2_, the culture medium in each well was replaced with a mixture composed of 90 μL of fresh medium and 10 μL of CCK-8 reagent (Beyotime, #C0038). The plate was incubated for 1 h, and the absorbance at 450 nm was measured using a SpectraMax iD multi-functional microplate reader from Molecular Devices. All procedures were conducted under sterile conditions to prevent microbial contamination and interference with the assay results.

### Transwell assay, scratch assay and tube formation assay

After confirming that the potential adverse effects caused by transduction and cell mixing have been eliminated, we first evaluated the invasion, migration and tube formation abilities of the cell mixtures outside the chip model. For the Transwell assay, a 24-well Transwell plate was used (Corning, #3422). MSCs, Lv-GFP-MSCs and Lv-Slit3-MSCs were separately seeded in the lower compartment at a density of 5 × 10^3^ cells/cm^2^, and HUVECs were seeded in the upper compartment at the same time. After 24 h of culture at 37°C with 5% CO_2_, the inserts were removed. Cells that had migrated to the lower surface of the membrane were fixed with 4% paraformaldehyde for 20 min, and stained with 0.1% crystal violet solution (Solarbio, #G1063) for 15 min. Five random fields at 200× magnification were selected, and the number of migrated cells was counted to evaluate the migratory capacity of endothelial cells. For the scratch assay, HUVECs were seeded in the lower compartment of a 6-well co-culture system (Corning, #3450) at 5 × 10^3^ cells/cm^2^, while MSCs, Lv-GFP-MSCs or Lv-Slit3-MSCs were seeded separately in the upper compartment. When the HUVECs monolayer reached over 90% confluence, a linear scratch was created using a 200 μL sterile pipette tip. Cells were gently washed with PBS to remove debris and then cultured with a fresh mixture of DMEM and α-MEM. Images of the scratch area were captured at 0 and 6 h under an inverted microscope. The wound area was quantified using ImageJ software, and the cell migration rate was calculated to assess the directional motility. For the tube formation assay, HUVECs were resuspended at a density of 1 × 10^4^ cells per well in conditioned medium obtained from Lv-Slit3-MSCs or from MSCs, which served as the experimental and control groups, respectively. The cell suspensions were then seeded onto type I collagen-coated surfaces and incubated at 37°C for 12 h. Capillary-like structures were subsequently visualized under a 10× objective. Total tube length, number of branch points and number of loops were quantified using the Angiogenesis Analyzer plugin in ImageJ software to evaluate the *in vitro* angiogenic capacity of endothelial cells.

### Microfluidic chip design, mold preparation and chip fabrication

After the initial functional evaluation of the cells outside the chip model, we fabricated the microfluidic chips and performed on-chip cultures and assessments. This study employed standard soft lithography technology to customize a 3D microfluidic chip for investigating osteo-angiogenic interaction. Briefly, the chip was designed with AutoCAD 2024, and the design file was used to manufacture high-resolution photolithography masks by Shenzhen Anme Electrical Technology. Following the soft-photolithography protocol, the silicon wafer was sequentially cleaned with acetone, isopropanol and water, and then dried with a nitrogen stream. After drying on a hot plate in a dust-free room, SU-8 3050 negative photoresist (SU-8, Kayaku, #Y311075 0500L1GL) was poured onto the wafer. The wafer was held by vacuum on a spin coater (Laurell, #WS-650-23B). The wafer was spun at 500 rpm for 10 s followed by 1500 rpm for 30 s. The coated wafer was soft-baked on a hot plate at 65°C for 5 min and then at 95°C for 10 min. Subsequently, the wafer was exposed to UV light through the photomask for 10 s in a mask aligner from ABM, Inc., USA. The exposed wafer was developed by applying developer solution (Thermo Scientific, #413900050) for 15 min, followed by rinsing with isopropanol and drying with nitrogen. Finally, a hard bake was performed on a hot plate at 120°C for 30 min to complete fabrication of the SU-8 mold (Supplementary Figure S1).

To fabricate the polydimethylsiloxane (PDMS) chip, the SYLGARD 184 silicone elastomer base (SYLGARD™ 184 Silicone Elastomer Kit, #01673921) was thoroughly mixed with the curing agent at a weight ratio of 10:1 for 2 min. The mixture was poured onto the SU-8 mold and degassed in a vacuum desiccator until all air bubbles were removed. After thermal curing at 65°C overnight, the solidified PDMS block was carefully peeled from the mold and trimmed to an appropriate size. Fluidic inlets and outlets were created by a 1-mm punch (Integra Miltex, #33-31AA). The structured PDMS chip and 75 × 25 mm glass slides were simultaneously treated with oxygen plasma in a plasma cleaner (HARRICK PLASMA, #PDC-32G-2), and then brought into conformal contact to achieve irreversible bonding. The assembled chip was sterilized by UV irradiation in a biosafety cabinet for 30 min before cell seeding.

### Hydrogel design and injection optimization

We designed a three-microchannel chip platform. In the middle tissue channel, a porous hydrogel containing a mixture of HUVECs and hMSCs was introduced. Two adjacent medium channels supplied nutrients to the tissue channel via a microfluidic pump, thereby mimicking physiologically relevant fluid circulation. At the interface between the tissue channel and the medium channels, micro-trapezoidal barriers with a gap of 20 μm were fabricated to prevent cells and hydrogel from invading the medium channels. However, preliminary tests revealed that hydrogel inside the tissue channel underwent marked degradation within 7 days of culture. To overcome this limitation, we first compared several hydrogel formulations and then developed a pre-assembled collagen microfiber strategy. A porcine type-I collagen solution at 4 mg/mL (Chondrex, #1203) was mixed with 5× DMEM and reconstruction buffer (0.05 mol/L NaOH, 200 mmol/L HEPES and 0.26 mol/L NaHCO_3_) at a volume ratio of 7:2:1. The mixture was incubated at 37°C overnight to allow the components to completely self-assemble into a gel [9–11]. The resulting gel was mechanically fragmented into microfiber pieces smaller than 100 μm, mixed with the cell suspension and centrifuged at 3000 rpm for 10 min to remove excess fluid, yielding a dense, uniformly distributed microfiber–cell composite. The fiber density after centrifugation was calculated to be approximately 7 mg/mL. The composite was then injected into the tissue channel, where it formed a mechanically stable 3D matrix that supported cell migration and angiogenesis after 7 days of culture in a mixed medium consisting of DMEM and α-MEM. This approach enabled stable vascularized bone formation within the chip, outperforming rapid physiological fibrinogen/thrombin hydrogel [12, 13], photo-controlled spatial cross-linking of gelatin methacryloyl (GelMA) [14, 15] and traditional mechanical reinforcement of collagen microfibers with respect to degradation. All procedures were performed under sterile conditions with pre-cooling to 4°C to preserve cell viability. The pore size, stiffness and rheological properties of the hydrogels were quantitatively calibrated to ensure batch-to-batch consistency.

### Microfluidic culture system

To simulate the dynamic interstitial fluid flow present in the bone microenvironment *in vitro* and to evaluate its biological effects, this study compared the cellular functions under dynamic perfusion and static culture conditions. All samples were randomly assigned to two groups, the dynamic perfusion group, in which culture medium was continuously delivered by a microfluidic pump, and the static control group, in which medium was replaced every 48 h according to standard culture practice. A 5 mL sterile syringe (Terumo, #SS + 05L) served as the medium reservoir and was fitted with a 0.6-mm sterile needle (Terumo, #AN*2325R1) inside a laminar flow hood. The assembled perfusion system was connected to the chip inlets and outlets under sterile connections to ensure sealed interfaces. A four-channel single-push mode infusion pump (LongerPump, #LSP04-1A) was used to precisely maintain a perfusion rate of 40 μL/h. This flow rate, calibrated in pilot experiments, generated a laminar shear stress of approximately 0.5–1 dyn/cm^2^ within the channel, a range intended to mimic the physiological shear experienced by cells in the bone microvasculature. The entire system was placed in a humidified incubator at 37°C with 5% CO_2_ to maintain stable culture conditions. After several days of continuous culture, samples from both of the groups were harvested simultaneously. Cell samples were collected for subsequent immunofluorescence staining, and the conditioned media were collected for cytokine secretion profiling.

### Biodegradability evaluation

To systematically evaluate the hydrogel degradation within the microfluidic chip platform, this study was designed to quantify the degradation characteristics through temporal changes in the morphological structure of the materials. Chip samples from each group were placed in a humidified incubator at 37°C with 5% CO_2_ and continuously cultured for 1, 4, 7, 14 and 21 days. The pre-warmed culture medium was completely replaced every 48 h to maintain nutrient supply and remove degradation products. At each predetermined time point, images were systematically acquired using an inverted fluorescence microscope (Invitrogen, #AMF5000) with 4×, 10× and 20× objectives. Qualitative evaluations encompassed two principal aspects, namely morphological alterations, which comprised temporal changes in fiber architecture and surface topography, and structural integrity, which was assessed by the regularity of material edges and the extent of overall contraction. All procedures were performed under aseptic conditions to avoid interference from microbial contamination. Image analysis was carried out using ImageJ software, and the results were independently assessed by two researchers.

### Immunofluorescence staining

After the stability of the hydrogel-cell composite in the tissue channel was confirmed within 21 days, immunofluorescence staining was performed to evaluate morphology and osteogenic differentiation. Samples were gently rinsed twice with pre-warmed PBS to remove residual culture medium. A 4% paraformaldehyde solution (Biosharp, #BL539A) was perfused into the microchannels and left to fix overnight at 4°C. Following PBS rinses, the samples were permeabilized with 0.5% Triton X-100 (Beyotime, #P0096) for 15 min at room temperature. Immunofluorescence blocking solution (Beyotime, #P0102) containing 5% normal goat serum was applied for 60 min at room temperature. For detection of RUNX2, mouse anti-RUNX2 monoclonal antibody (Mouse anti-RUNX2, Abcam, #AB76956, diluted 1:250 reacted with hMSCs) diluted with PBS was introduced into the microchannels and incubated with gentle agitation overnight at 4°C. After thorough washing, the corresponding Alexa Fluor 488-conjugated secondary antibodies (Invitrogen, goat anti-mouse, #A32723, diluted 1:1000 reacted with hMSCs) were added. The secondary antibody incubation was carried out in the dark for 45 min at room temperature. Actin-Tracker Red (Beyotime, #C2205S, diluted 1:200) was applied for 60 min to visualize the cytoskeleton. Hoechst 33342(Invitrogen, #H3570, diluted 1:2000) was used for nuclear counterstaining for 10 min. All staining steps were followed by a final PBS rinse.

### ELISA assay

To test potential targets and biomarkers during osteogenic differentiation on the three-microchannel chip model, a highly sensitive enzyme-linked immunosorbent assay was performed. For the control group, conditioned medium was collected from hMSCs cultured in a 1:1 mixture of DMEM and α-MEM. For the experimental group, conditioned medium was harvested from Lv-Slit3-transduced hMSCs. On day 14 of culture, conditioned medium from each group was collected and immediately stored at −80°C until analysis. The following highly specific ELISA kits from Dumabio were used, including ALP (#DM4705), OPN (#DM5752), OCN (#DM5748), VEGF (#DM6751) and Agrin (#DM4822). All assays were performed according to the manufacturer’s instructions. An eight-point standard curve covering 0–500 ng/mL was established, with the correlation coefficient *R*^2^ of each standard curve >0.99, the intra-assay coefficient of variation <8% and the inter-assay coefficient of variation <12%. The measured concentrations were normalized to the total protein content of the conditioned medium.

### RT-qPCR

After successfully validating the potential target with human-derived cells, we further examined whether the Slit3–Agrin axis exhibited species-specific effects by conducting mechanistic studies with mouse-derived cells. In the subsequent mechanistic investigation, RT-qPCR was first performed to evaluate the mRNA expression. Total RNA was extracted from cells using the RNA-Quick Purification Kit (ESScience, #RN001) and reverse-transcribed into cDNA using the RT Master Mix for qPCR II (MedChemExpress, #HY-K0511A). The amplification reaction was carried out with 2 × SYBR Green qPCR Master Mix (Selleck, #B21202) on a Thermo Fisher Scientific QuantStudio 6 Flex real-time PCR system. Primers were designed and synthesized by Sangon Biotech Co., Ltd (Supplementary Figure S2). Gene expression levels were analyzed using the comparative threshold cycle method (2^−ΔΔCt^), with β-actin serving as the internal reference gene for normalization.

### Western blot assay

To further evaluate the protein expression levels of several osteogenic markers, a western blot assay was performed. Cells were lysed on ice for 30 min using RIPA lysis buffer (Beyotime, #P0039) supplemented with protease and phosphatase inhibitors. The lysate was centrifuged at 14 000 rpm for 15 min at 4°C, and the supernatant was collected as the total protein extract. The protein concentration of each sample was determined using the BCA method (Thermo Scientific, #23225). Equal amounts of protein were mixed with the loading buffer and heated at 95°C for 5–10 min to ensure complete denaturation. Proteins were separated on 8–12% gradient SDS-PAGE gels and transferred onto a PVDF membrane via wet transfer at 100 V for 90 min. The membranes were blocked and then incubated with the respective primary antibodies overnight at 4°C. The primary antibodies and their dilution ratios used were β-actin (1:10 000), Slit3 (1:1000), OPN (1:1000), Agrin (1:1000), RUNX2 (1:1000), β-catenin (1:1000) and Histone-H3 (1:1000). On the following day, the membrane was washed extensively with TBST and incubated with the appropriate HRP-conjugated secondary antibody (1:3000) for 1 h at room temperature. Protein bands were detected using ECL chemiluminescent reagents, and the chemiluminescent signals were captured with a ChemiDoc MP imaging system from Bio-Rad. The band intensities were quantified by gray-value analysis using the ImageJ software.

### Immunofluorescence staining, osteogenic staining, tube formation and scratch assay

To determine whether the osteogenic and angiogenic effects of Slit3 are primarily mediated through Agrin, we first compared the osteogenic and angiogenic capacities of Slit3 and Agrin, given that the pro-angiogenic function of Slit3 has been previously documented. For the immunofluorescence staining, BMSCs on day 5 were fixed, permeabilized and blocked with the same protocol as above. They were incubated with a primary antibody (Rabbit anti-RUNX2, #A11753, diluted 1:200 reacted with BMSCs) overnight, and then with a secondary antibody (Goat anti-Rabbit, #A11012, diluted 1:500) for 1 h followed by PBS rinses. Actin filaments and nuclei were subsequently labeled with Actin-Tracker and DAPI, and images were captured under a confocal microscope. For the ALP staining, cells were gently rinsed twice with PBS and fixed with 4% paraformaldehyde phosphate buffer (PFA, Biosharp, #BL539A) for 10 min at room temperature. After removal of the PFA solution, 200 μL of ALP working solution, prepared by dissolving 0.4 g ALP staining substrate powder in 100 mL ddH_2_O according to the kit instructions, was added to each well and incubated for 15 min at room temperature. Images were acquired with an inverted optical microscope to evaluate early osteogenic differentiation. For the Alizarin Red staining, following fixation with 4% paraformaldehyde, cells were washed twice with PBS and then stained with 200 μL of 0.4% Alizarin Red S solution for 5 min at room temperature to specifically label calcium deposits. After rinsing with deionized water, mineralized nodules were observed under a microscope to assess late-stage osteogenic differentiation and mineralization. The tube formation and scratch wound-healing assays were conducted as described in the preceding section. To evaluate whether Slit3 overexpression could promote angiogenesis and migration in the absence of Agrin, three groups were compared, including the untreated control group, the Ad-Slit3 group and the Ad-Slit3 + si-Agrin group.

### TOP Flash/FOP Flash and chromatin immunoprecipitation analysis

In the initial mRNA sequencing analysis, Slit3 was identified as a differentially expressed gene during osteogenic differentiation. Subsequently, combined omics analysis was performed to compare the OIM group with the OIM + Ad-Slit3 group, and the Wnt signaling pathway was found to be activated. Given the well-established role of the Wnt/β-catenin pathway as a master regulator of osteogenic differentiation and bone formation, we prioritized this pathway for validation once it emerged as significantly enriched in our Kyoto Encyclopedia of Genes and Genomes (KEGG) analysis. For the TOP/FOP Flash assay, cells were seeded in a 96-well plate at 5 × 10^4^ cells/cm^2^. The experimental group was transduced with Ad-Slit3, and the control group with Ad-GFP. Transfections were performed using Lipofectamine 3000 (Thermo Fisher, #L3000015) with 300 ng of TOP or FOP Flash reporter plasmid and 30 ng of pRL-SV40 per well. After 24 h, the medium was changed, and the cells were cultured for an additional 24 h. Cells were lysed in 200 μL of passive lysis buffer (Promega, #E1910) for 15 min, and the lysate was centrifuged at 12 000 rpm for 5 min at 4°C. To measure luciferase activities, 20 μL of supernatant was first mixed with 100 μL of LAR II reagent to record firefly luciferase activity at 560 nm, after which 100 μL of Stop & Glo reagent was added to measure Renilla luciferase activity at 480 nm. The TOP/FOP ratio was calculated as (TOP Flash firefly RLU/TOP Flash Renilla RLU)/(FOP Flash firefly RLU/FOP Flash Renilla RLU). A ratio greater than 2 indicated significant β-catenin activation, consistent with previous reports (Supplementary Figure S3). For the chromatin immunoprecipitation assay, cells were fixed with 1% formaldehyde for 10 min at room temperature, and cross-linking was quenched with 0.125 M glycine for 5 min. After washing with cold PBS, the cells were lysed on ice for 10 min. Nuclei were pelleted by centrifugation at 12 000 rpm for 5 min at 4°C and lysed in a nuclear lysis buffer. Chromatin was sonicated to fragments of 200–1000 bp and then centrifuged at 14 000 rpm for 10 min at 4°C, and the supernatant was collected. For immunoprecipitation, 50 μL of protein A/G beads (Beyotime, #P2078), blocked with 5% BSA and washed with TBSE buffer, were mixed with the chromatin and 2 μg of the target antibody. For the negative control, 2 μg of normal rabbit IgG was added. The mixtures were incubated overnight at 4°C with rotation. The beads were then added and incubated for an additional 2 h, followed by sequential washes with low-salt buffer, high-salt buffer, LiCl buffer and TE buffer. Immune complexes were eluted with 200 μL of elution buffer, and cross-links were reversed by incubation with 5 M NaCl. Samples were treated with RNase A and Proteinase K, and DNA was purified by ethanol precipitation and dissolved in RNase-free water.

### Animal experiments

To evaluate the osteogenic and angiogenic effects of potential targets in osteoporosis, we established an ovariectomized (OVX) mouse model. All animal experiments were conducted in accordance with the International Guide for the Care and Use of Laboratory Animals and were approved by the Research Ethics Committee of Chongqing Medical University, approval number IACUC-CQMU-2024-01004. Twenty 8-week-old specific-pathogen-free female C57BL/6 mice, weighing 16 ± 2 g, were purchased from the Experimental Animal Center of Chongqing Medical University and acclimatized for one week under standard conditions consisting of a 12-h/12-h light–dark cycle, constant temperature and humidity, and free access to food and water. A postmenopausal osteoporosis model was induced by bilateral ovariectomy. After the mice were anesthetized with 1% pentobarbital sodium, bilateral incisions were made on the back and both ovaries were completely excised. The incisions were then sutured layer by layer. In the sham operation group, the ovaries were only exposed but not removed. One week after surgery, the mice were randomly divided into five groups with four mice per group using a completely randomized design, including the sham operation group, the OVX group, the OVX + rAAV9-GFP group, the OVX + rAAV9-Slit3 group and the OVX + rAAV9-Slit3 + rAAV9-amiR-Agrin group. Each treatment group received a single tail-vein injection of the corresponding AAV serotype 9 at a dose of 5 × 10^11^ viral particles per mouse. After 8 weeks of intervention, the mice were deeply anesthetized and euthanized by CO_2_ inhalation. Immediately after euthanasia, the femurs were harvested.

### Micro-CT and histological analysis

To evaluate the osteogenic effect of the potential target, micro computed tomography (micro-CT) and histological analyses were performed. For the hematoxylin and eosin staining, tissue samples were rinsed three times with PBS and fixed in 4% paraformaldehyde at 4°C overnight. The samples were dehydrated by sequential immersion in 70%, 80% and 90% ethanol for 1 h each, and then in two changes of 99.5% ethanol for 30 min each. After dehydration, the samples were infiltrated in molten paraffin at 55–57°C for 6 h and subsequently embedded in paraffin blocks at 4°C overnight. Sections were cut at 5–7 μm thickness using a microtome and stored in 5% ethanol solution to prevent shrinkage. The sections were dried at room temperature for 3 h, deparaffinized in xylene for 10 min and rehydrated through a descending series of 100%, 90% and 70% ethanol, each for 2 min. The sections were stained in hematoxylin solution for 10 min, rinsed in running water for 10 min and then counterstained in eosin solution for 3 min. After staining, the sections were dehydrated through 70%, 90% and 100% ethanol for 2 min each, cleared in xylene and mounted with neutral resin for microscopic observation. For Masson’s staining, deparaffinized and rehydrated sections were first stained with Weigert’s iron hematoxylin for nuclei. After rinsing, the sections were stained with a Ponceau-acid fuchsin solution to label the cytoplasm and muscle fibers. The sections were then differentiated and mordanted in a phosphomolybdic–phosphotungstic acid solution for 5–10 min. Without rinsing, they were directly transferred to aniline blue solution for 5–10 min to stain collagen fibers blue. After a brief rinse, the sections were differentiated in 1% acetic acid for 1–2 min to enhance contrast. Finally, the sections were dehydrated rapidly through ethanol, cleared in xylene and mounted with neutral resin. The distribution and content of collagen fibers in the bone tissue were observed under a light microscope.

### Statistical analysis

All analytical procedures followed internationally recognized statistical standards. Continuous variables were expressed as mean ± standard deviation. The Shapiro–Wilk test was used to assess the normality of data distributions. Statistical methods were selected according to experimental design and data type, for example, Student’s *t*-test was used for comparisons between the two groups when normality and homogeneity of variance were satisfied. One-way ANOVA was used for multiple groups under similar assumptions. The Kruskal–Wallis test was used as a non-parametric alternative when those assumptions were violated. Two-way ANOVA was for experiments with two factors, and Scheirer–Ray–Hare non-parametric two-way ANOVA was for ranked data in a two-factor design. When an omnibus test yielded a significant result, *post hoc* comparisons were performed using Tukey’s honestly significant difference test following ANOVA, or Dunn’s test following the Kruskal–Wallis test. Statistical significance was defined as *P* < 0.05, with individual levels denoted by *P < 0.05, **P < 0.01, ***P < 0.001 and ****P < 0.0001. All analyzes were completed using R statistical software version 4.5.0.

## Results

### Identification of Slit3 as a potential target in BMSC osteogenesis by mRNA sequencing

mRNA transcriptome sequencing was performed on the OIM group and the control group, and a systematic analysis was conducted (Figure 2A). This analysis identified 333 significantly differentially expressed genes, of which 142 were upregulated and 191 were downregulated (Figure 2B). Among these, Slit3 exhibited a pronounced expression change that attracted our particular interest. A review of the literature indicated that Slit3, a secreted glycoprotein, plays a key role in the regulatory network coupling angiogenesis and bone development. However, its specific function in osteoblast differentiation remains unclear, and direct mechanistic evidence is still lacking. Therefore, we performed Western blot assay to determine the expression of Slit3 from day 1 to day 9. The results demonstrated that Slit3 protein expression began to increase significantly on day 3 and continued to rise through day 9, a temporal profile which is consistent with the key period of osteogenic differentiation (Figure 2C). KEGG pathway analysis further showed that the TGF-β signaling pathway was significantly activated, a pathway known to play a central role in coordinating bone formation and angiogenesis (Figure 2D). Gene Ontology (GO) enrichment analysis of the differentially expressed genes revealed significant enrichment in biological processes such as osteoblast differentiation, extracellular matrix organization and regulation of vascular endothelial cell proliferation (Figure 2E). These findings indicate a close association between Slit3 expression and osteogenic differentiation. By integrating mRNA sequencing with experimental validation, we delineated the expression pattern of Slit3 during osteogenic differentiation.

**Figure 1:**
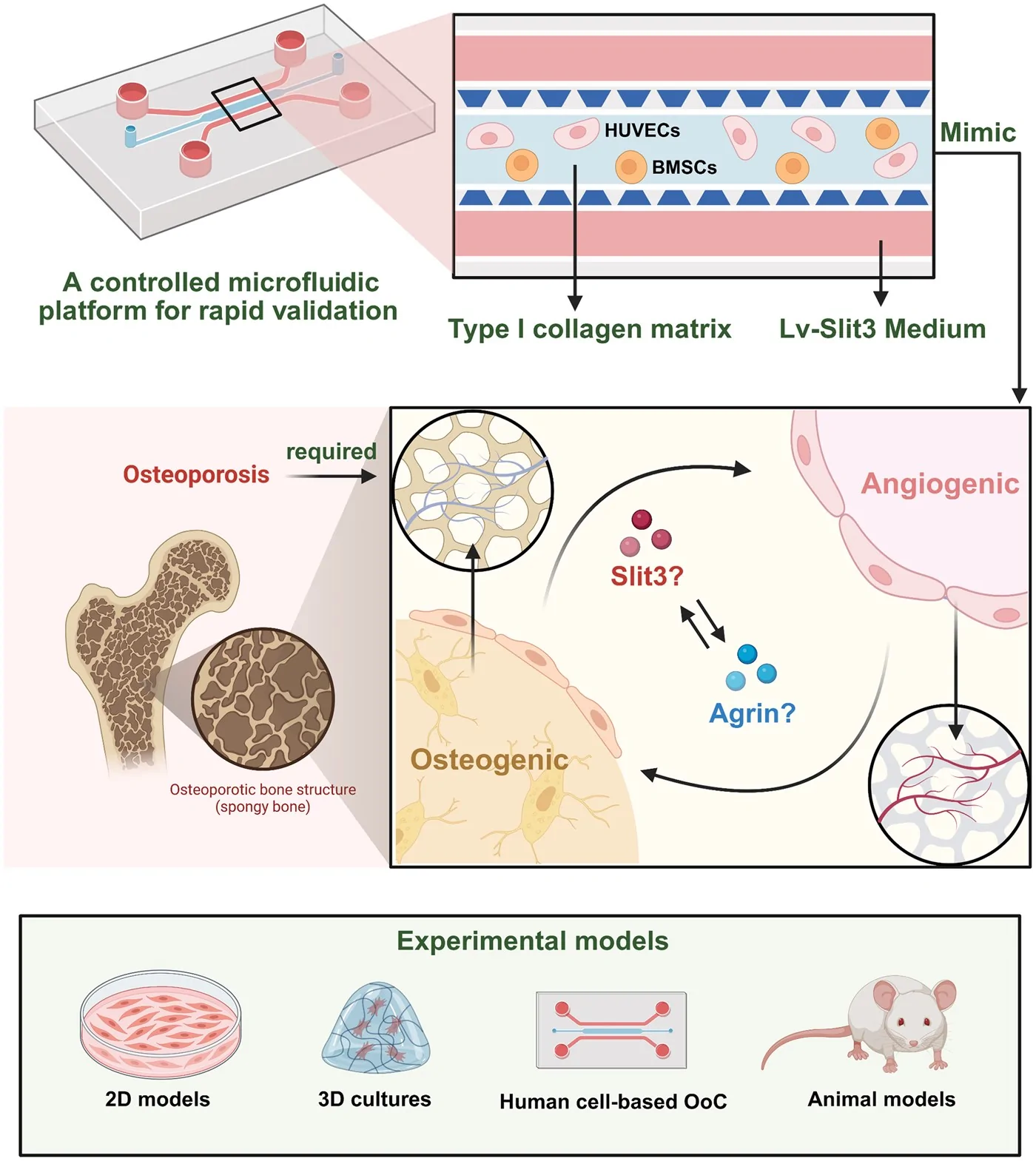
The general schematic diagram of a controlled microfluidic platform used HUVECs, BMSCs, Lv-Slit3 Medium and Type I collagen matrix to mimic the osteogenic and angiogenic interaction in relatively physiological environment of bone. The aim is to rapidly vaildate the Slit3-Agrin axis. The experimental models altogether include 2D models, 3D cultures, Human cell-based OoC and Animal models. Lv, HUVECs, BMSCs and OoC denote lentivirus, HUVEC, BMSCs and OoC, respectively.

**Figure 2:**
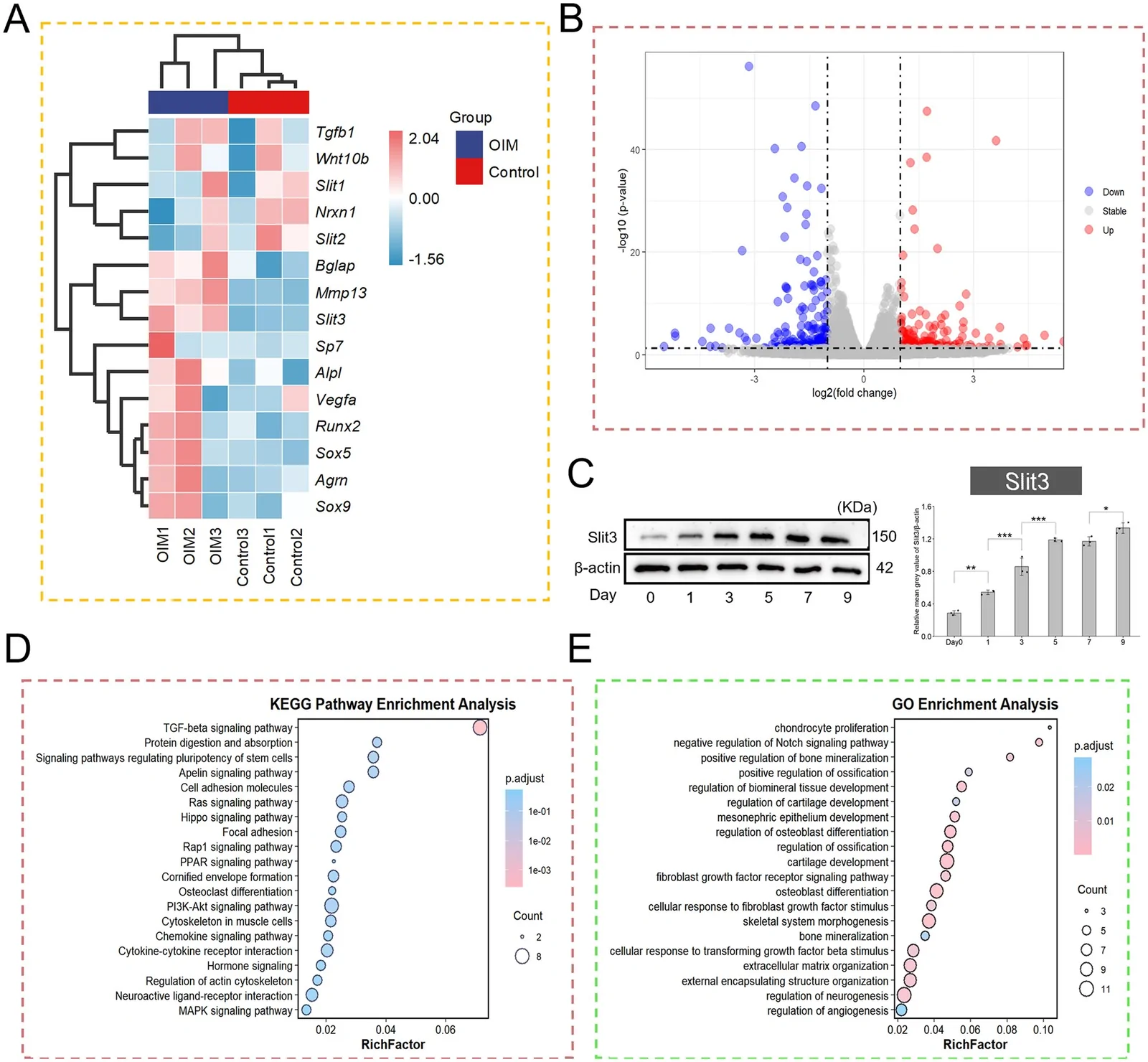
mRNA sequencing identified Slit3 during the osteogenic differentiation of BMSCs. (**A**) The top 20 significantly differentially expressed genes between the OIM group and the control group. (**B**) Volcano plot of gene expression during osteogenic induction. The *x*-axis represents log_2_(fold change), and the *y*-axis represents − log_10_(*P*-values). The numbers of upregulated and downregulated genes are indicated. (**C**) Expression of Slit3 during OIM induction from day 1 to day 9. (**D**) KEGG enrichment analysis of differentially expressed genes, identifying significantly enriched biological pathways. The enrichment score indicates the importance of pathway relevance. (**E**) GO enrichment analysis showing the number of differentially expressed genes in each GO term. The enrichment score reflects the statistical significance of GO term enrichment.

**Figure 3:**
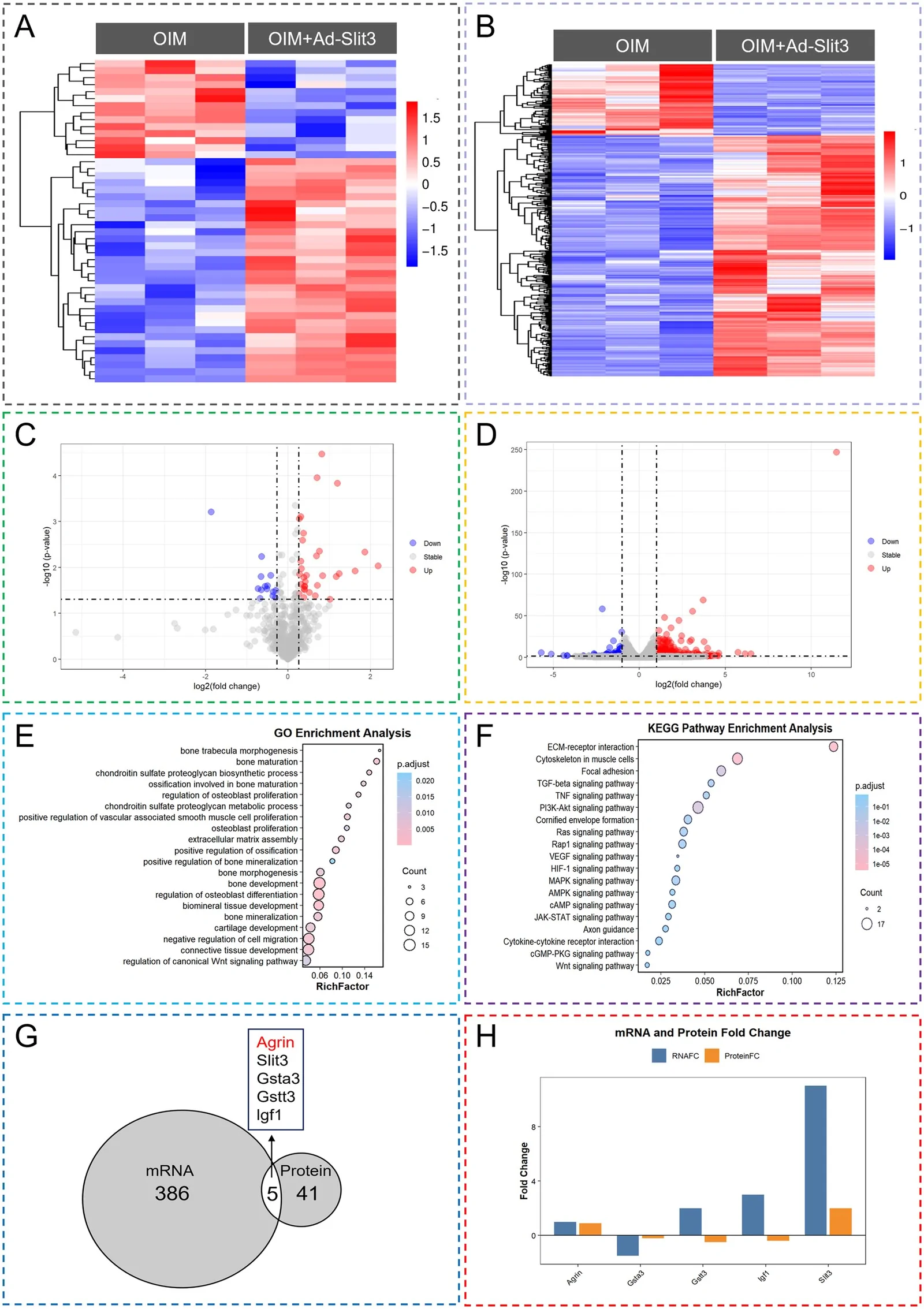
Combined analysis of mRNA and secretory protein sequencing. (**A**) Secreted protein profiling in the OIM group and the OIM + Ad-Slit3 group. (**B**) mRNA sequencing in the OIM group and the OIM + Ad-Slit3 group. (**C**) Volcano plots of secreted protein sequencing. (**D**) Volcano plots of mRNA sequencing. The *x*-axis represents the log_2_(fold change) of expression between the two groups, and the *y*-axis represents –log_10_(*P*-values), indicating the statistical significance of differential expression. (**E**) GO enrichment analysis of differentially expressed mRNAs. The size of bubble shows the count of genes annotated to each GO term, and the enrichment score reflects the significance of GO term enrichment. (**F**) KEGG enrichment analysis of differentially expressed mRNAs, identifying significantly enriched biological pathways. The enrichment score indicates the importance of pathway relevance. (**G**) Combined analysis of mRNA and secretory protein sequencing. (**H**) Quantitative comparison between mRNA expression changes and secreted protein level changes.

**Figure 4:**
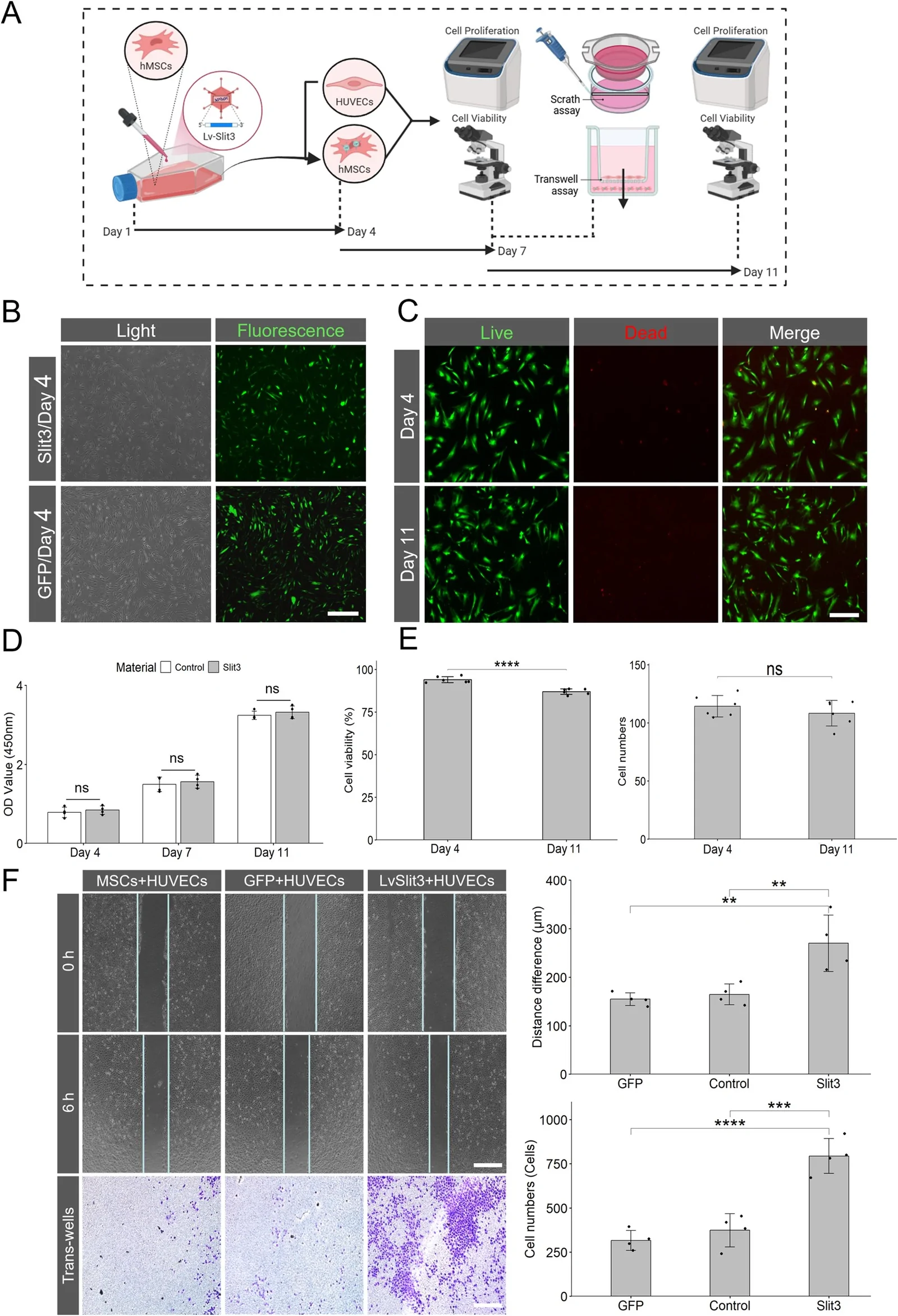
Pre-experiments of the chip platform. (**A**) Schematic diagram of the transduction procedure and timeline. (**B**) Fluorescence staining of hMSCs transduced with Lv-Slit3 or Lv-GFP on day 4. Scale bar = 200 μm. (**C**) Cell viability measured on days 4 and 11 in the Lv-Slit3 group. Scale bar = 100 μm. (**D**) Quantitative analysis of OD value in cck-8 assay on days 4, 7 and 11. Statistical comparisons were performed by nonparametric two-way ANOVA. (**E**) Quantitative analysis of cell viability and cell density on days 4 and 11 by ImageJ. Statistical comparisons were performed by Student’s *t*-test. (F) Scratch and Transwell assays, performed in the Lv-Slit3, Lv-GFP and control groups at 0 and 6 h. Scale bar = 200 μm. Statistical comparisons were performed by one-way ANOVA.

**Figure 5:**
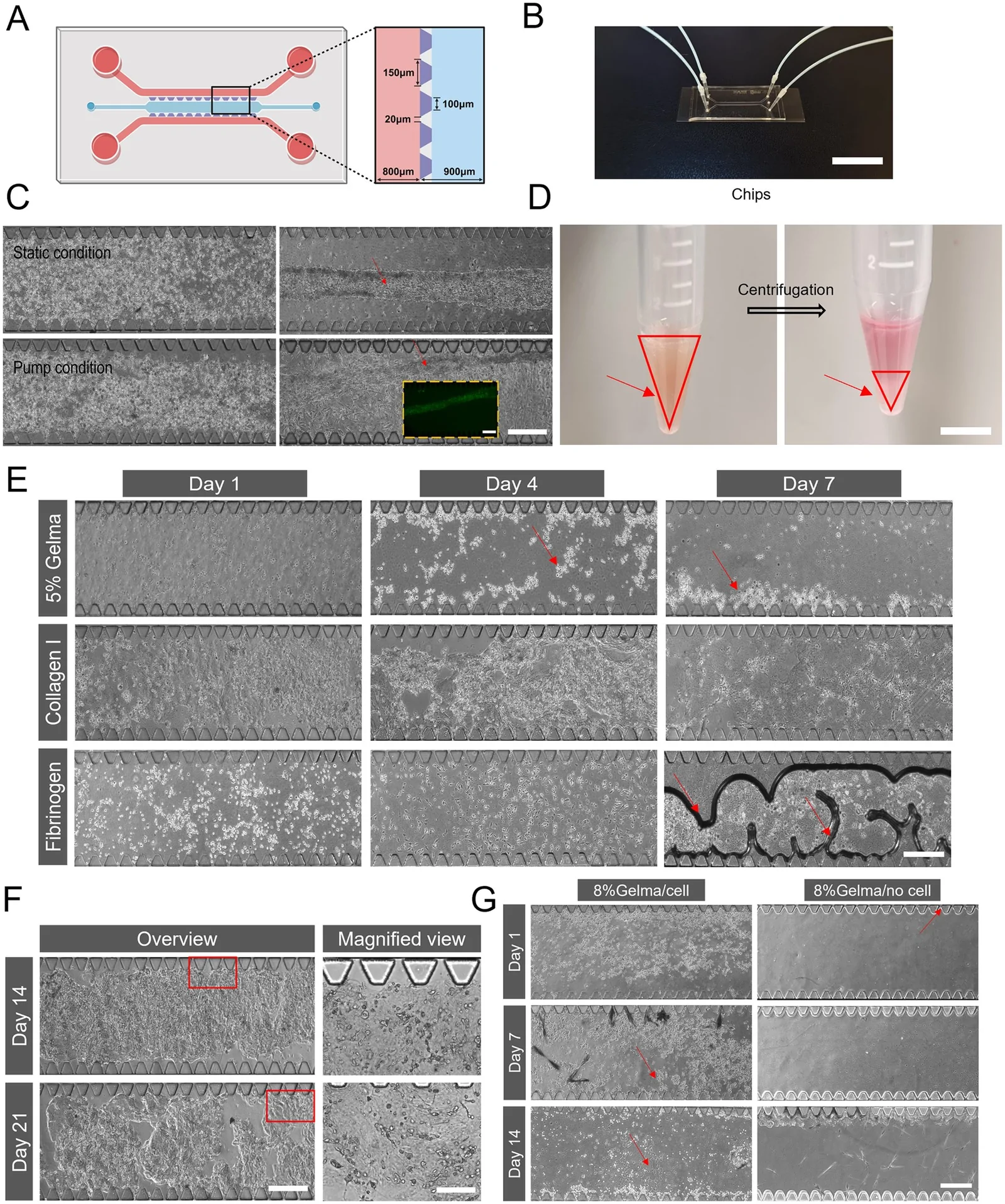
Chip design and feasibility tests. (**A**) Proportional diagram of the chip design. (**B**) Macroscopic observation of the chips. Scale bar = 2 cm. (**C**) Preliminary test of tissue channels under static and pump conditions. Yellow box indicated Calcein-AM/PI staining of bundle-like hydrogel matrix. Scale bar = 500 μm. (**D**) Comparison between self-assembly and compressed type-I collagen matrix. Red triangles indicate the collagen fibers complex. Scale bar = 1 cm. (**E**) Biodegradation test of different hydrogels on days 1, 4 and 7. Scale bar = 500 μm. (**F**) Microscopic observation of compressed type-I collagen at 4× and 10× (red rectangle) magnification on days 14 and 21. Scale bar = 500 μm. (**G**) Biodegradation test of the 8% GelMA group with or without cells on the days 1, 7 and 14. Scale bar = 500 μm.

**Figure 6:**
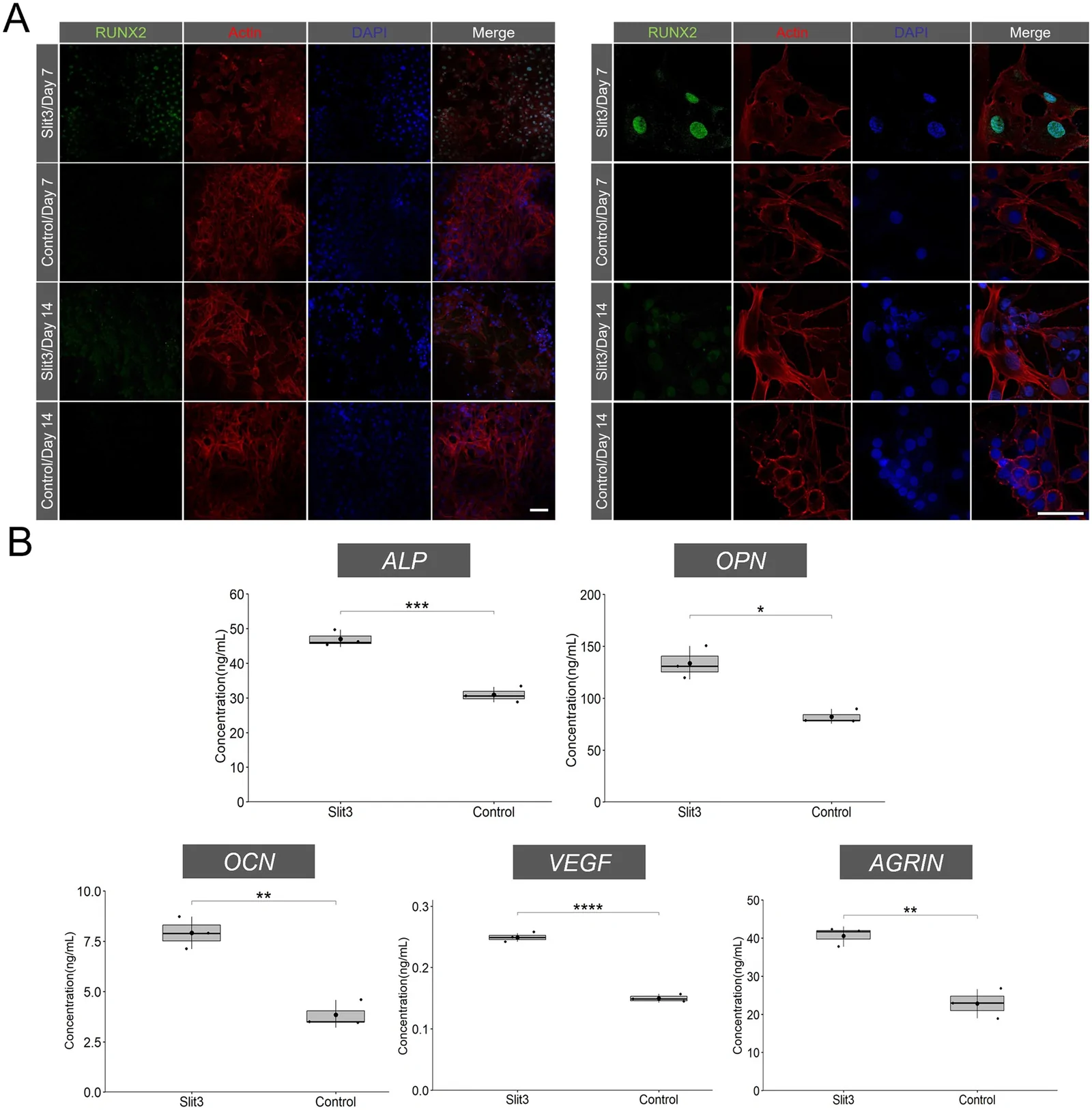
Immunofluorescence staining and biomarkers testing on chips under pump condition. (**A**) Immunofluorescence staining of RUNX2, ACTIN and DAPI on tissue channels under 10× and 86× magnification on STED 8. Scale bar = 100 and 5 μm, respectively. (**B**) ELISA detection of biomarkers including ALP, OPN, OCN, VEGF and AGRIN on the day 14. Statistical comparisons were performed by Student’s *t*-test.

**Figure 7:**
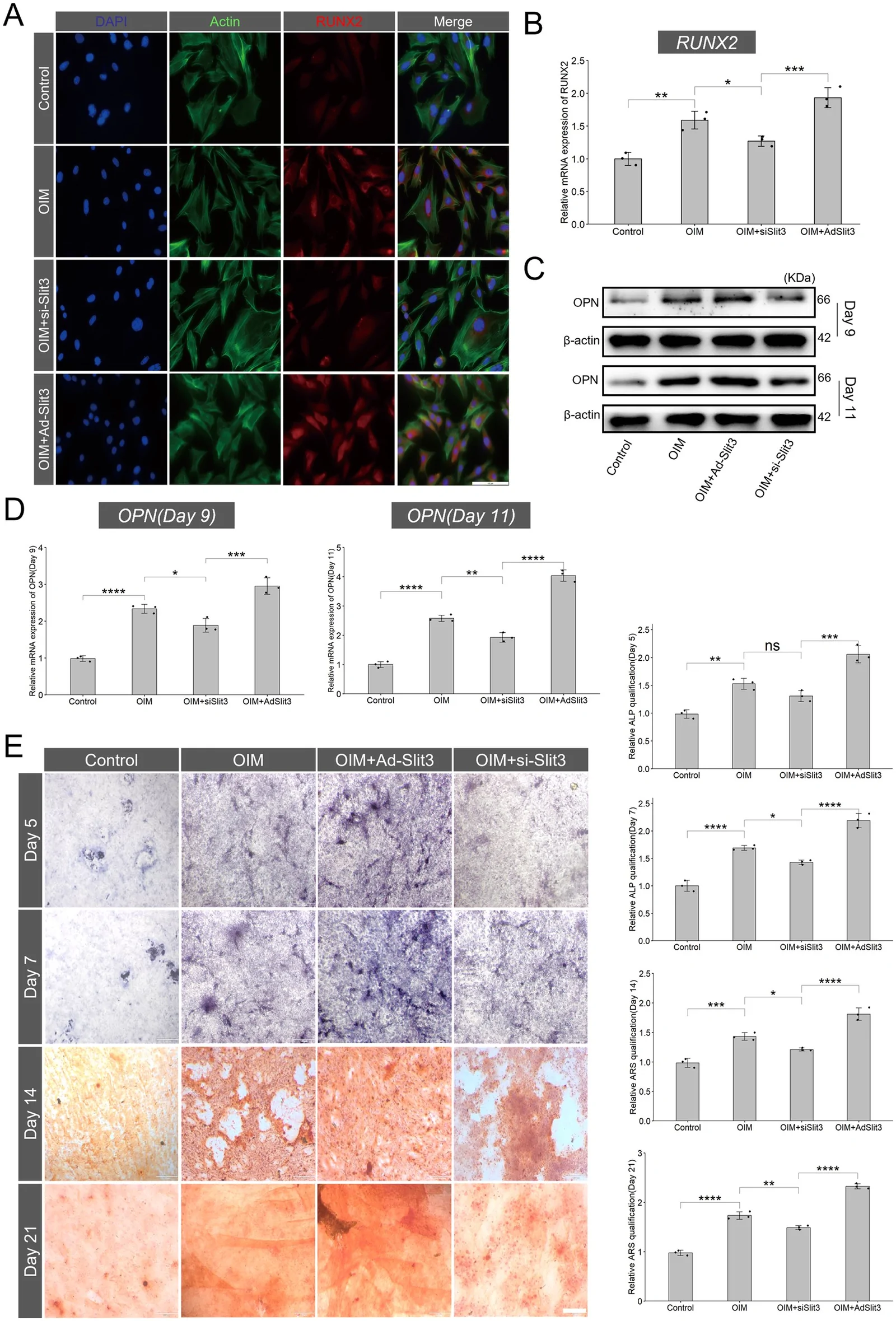
The osteogenic effect of Slit3 was verified *in vitro* through immunofluorescence staining, Western blotting, RT-qPCR, ALP and Alizarin red staining. (**A**) Immunofluorescence staining of RUNX2, ACTIN and DAPI on day 5. Scale bar = 10 μm. (**B**) RT-qPCR detection of RUNX2. Statistical comparisons were performed by one-way ANOVA. (**C**) Western blotting detection of OPN expression on days 9 and 11. (**D**) RT-qPCR detection of OPN. Statistical comparisons were performed by one-way ANOVA. (**E**) Qualitative and quantitative analysis of ALP and Alizarin red staining on the days 5, 7, 14 and 21. Scale bar = 100 μm. Statistical comparisons were performed by one-way ANOVA.

**Figure 8:**
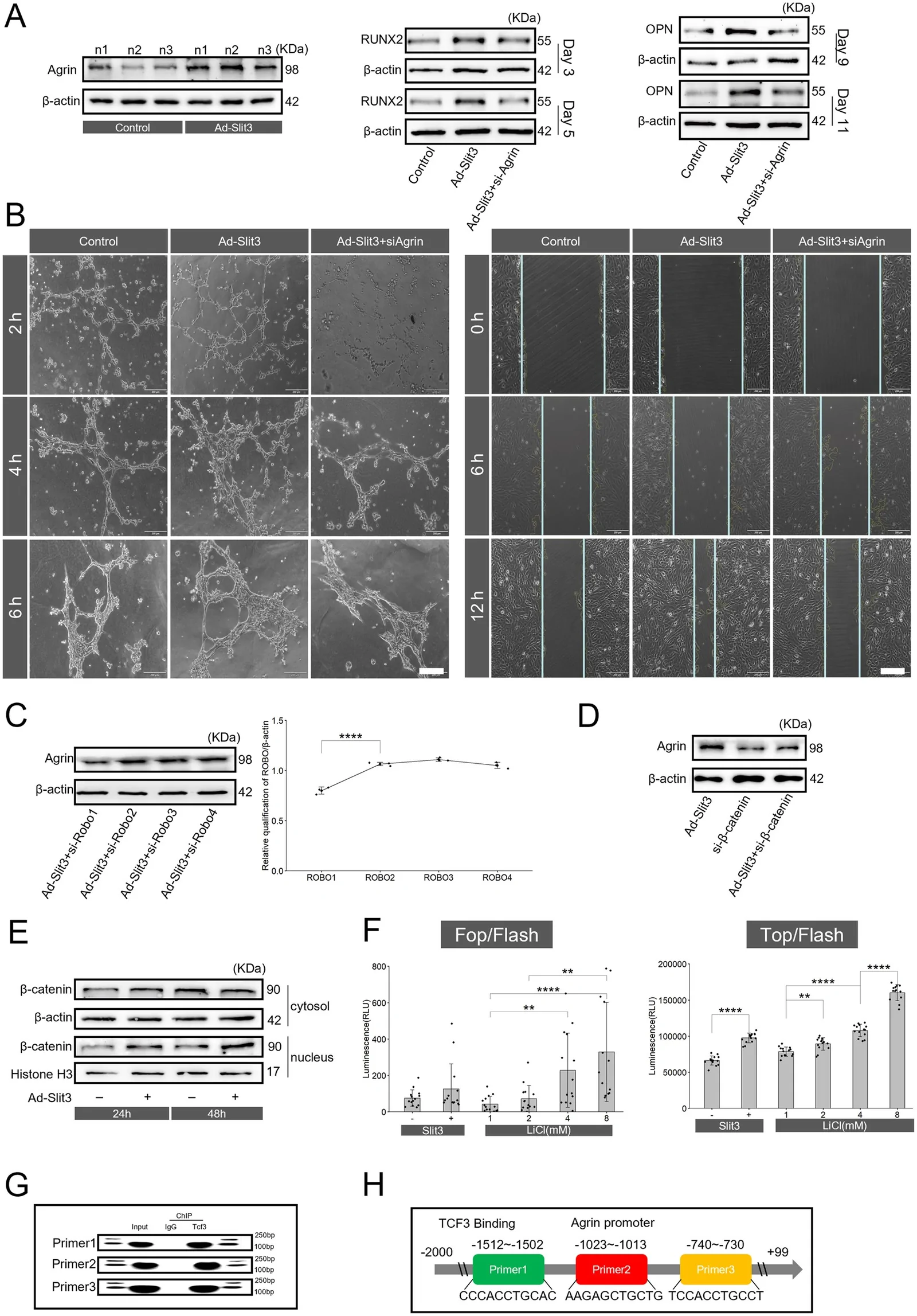
Agrin promotes osteogenesis in bone and the regulatory mechanisms. (**A**) Western blotting detection of Agrin, RUNX2 and OPN protein expression. (**B**) Tube formation and stretch assay of the control, Ad-Slit3 and Ad-Slit3 + si-agrin groups. Scale bar = 200 μm. (**C**) Western blotting detection of Robo1 to Robo 4 expressions. Statistical comparisons were performed by one-way ANOVA. (**D**) Western blotting detection in the Ad-Slit3, si-β-catenin, and Ad-Slit3 + si-β-catenin groups. (**E**) Western blotting detection of Slit3 that promotes β-catenin’s nuclear translocation. (**F**) Fop/Flash and Top/Flash reporter assays. Statistical comparisons were performed by Kruskal–Wallis test and one-way ANOVA. (**G**) ChIP detection using TCF3 primer. (**H**) Diagram of TCF3 binding sequence.

### Multi-omics identification of Agrin as a Slit3 downstream effector

Based on the previous identification of Slit3 as a potential regulator of osteogenic differentiation in BMSCs, we performed integrated transcriptomic and proteomic analyses to systematically reveal the downstream signaling network coordinated by Slit3, and we identified Agrin as a key downstream effector (Figure 3A and B). At the proteomic level, 46 differentially expressed proteins were identified, with 32 upregulated and 14 downregulated (Figure 3C). At the mRNA level, 391 differentially expressed genes were detected, of which 302 were significantly upregulated and 89 were significantly downregulated (Figure 3D). GO enrichment analysis further revealed that the differentially expressed genes were predominantly involved in biological processes such as cell differentiation, developmental processes and extracellular matrix organization (Figure 3E). KEGG enrichment analysis indicated that these differentially expressed genes were significantly enriched in pathways closely related to bone metabolism, including the PI3K-Akt, Wnt, Ras and estrogen signaling pathways (Figure 3F). Through integrated analysis of the transcriptomic and proteomic data, five molecules, namely Slit3, Agrin, Gsta3, Gstt3 and Igf1, showed consistent alterations at both the mRNA and protein levels (Figure 3G), suggesting that their expression may be transcriptionally regulated by Slit3. Quantitative comparison demonstrated that Agrin expression was significantly upregulated by Slit3 at both the mRNA and protein levels (Figure 3H), which indicated that Agrin may function as a critical downstream effector in the Slit3 signaling pathway.

### Assessment of cell viability and function during transduction and co-culture

To rapidly confirm the findings, we first established a three-microchannel chip model as an intermediary platform to test the Slit3–Agrin axis using human-derived cells. Before functional verification, we constructed stable Slit3-overexpressing hMSC lines with lentiviral vectors to ensure long-term transgene expression (Figure 4A). The light and fluorescence microscopic observations indicated that hMSCs were successfully transduced with Slit3 or GFP (Figure 4B). Calcein-AM/PI double staining confirmed that cells maintained high viability on days 4 and 11 (Figure 4C). CCK-8 assays further indicated that the transduction of Slit3 did not affect cell viability (Figure 4D). The cell density and the cell viability (93.94 ± 1.79% and 86.99 ± 1.63%, respectively) remained stable from day 4 to day 11 (Figure 4E). Furthermore, the scratching assay showed that the migration distance of HUVECs in the Lv-Slit3 group (269.95 ± 58.15 μm) was significantly greater than that in the Lv-GFP group (154.6 ± 13.1 μm) (Figure 4F). The Transwell migration assay confirmed that the number of migrated cells in the Lv-Slit3 group (793 ± 98 cells) was significantly higher than that in the blank control group (373 ± 95 cells) and the Lv-GFP group (316 ± 57 cells) (Figure 4F). We then quantitatively assessed the angiogenic capacity of HUVECs (Supplementary Figure S4). Compared with the control medium group, the Lv-Slit3 group formed a more complex and denser tubular network on the type I collagen matrix, as demonstrated by a significant increase in the number of branch points (48 ± 9 numbers) and a significant extension of the total tube length (327 ± 52 μm).

### Optimization of the extracellular matrix hydrogel via collagen centrifugation

Using standard soft lithography, we fabricated PDMS chips with precise geometric features. The channel height was 100 μm, the lengths were 100 and 150 μm and the channels were separated by stepped micro-columns with a gap of 20 μm (Figure 5A and B). We then constructed a 3D microenvironment based on porcine type I collagen within the chip and evaluated it under both static and pump-driven culture conditions. In initial experiments, under both static and dynamic conditions, the collagen matrix spontaneously contracted into a denser fiber network within 3 days (Figure 5C). Notably, dynamic culture conditions accelerated fluid loss and exacerbated structural contraction. Calcein-AM/PI staining confirmed that cells remained highly viable throughout, indicating that the contraction process did not impair cell survival. To overcome collagen contraction, we developed a centrifugal compression strategy (Figure 5D). By applying a centrifugal force of 3000 rpm to the self-assembled collagen matrix, we actively removed excess water and formed a high-density collagen microfiber composite. This pre-compression treatment allowed the collagen to maintain long-term structural stability after injection into the microchannel, effectively preventing deformation or blockage caused by spontaneous contraction during culture. To comprehensively evaluate hydrogel stability in the chip environment, we performed biodegradation tests. The results showed that 5% GelMA and fibrinogen/thrombin composite gels underwent structural fragmentation and degradation as early as day 4 to day 7, whereas the centrifugally compressed type I collagen group maintained an intact, interconnected network (Figure 5E). Besides, the centrifugally compressed type I collagen group demonstrated satisfactory structural stability over a 21-day culture period (Figure 5F). The collagen network remained fully interconnected up to day 14. By day 21, although the structure began to develop a porous appearance, overall integrity was still preserved. We further explored the cause of GelMA fragmentation and found that the presence of cells markedly accelerated GelMA degradation (Figure 5G). In the cell-laden GelMA group, cell migration and proliferation likely exerted mechanical stress on the gel structure, leading to fragmentation, whereas the acellular GelMA group retained a relatively intact structure. In addition, cells in the GelMA group exhibited a non-polarized, dendritic morphology, suggesting that this matrix environment may delay the expression of normal physiological functions.

### The measurement of osteo-angiogenic biomarkers in the chip platform

After successfully constructing and optimizing the three-microchannel chip model, we systematically evaluated the effect of overexpression of Slit3 on vascular network formation and osteogenic differentiation within the centrifugated type I collagen matrix. We monitored the expression dynamics of the key transcription factor RUNX2 during early osteogenic differentiation on the chip. RUNX2 displayed pronounced nuclear accumulation on day 7, indicating successful activation of the osteogenic program (Figure 6A). By day 14, the RUNX2 signal had declined, consistent with the temporal regulatory pattern observed during osteogenic differentiation *in vivo*. To further quantify the osteo-inductive effect of Slit3, we used ELISA to measure multiple biomarkers in the chip effluents. On day 14, the concentrations of key osteogenic and angiogenic factors in the Lv-Slit3 group were significantly higher than those in the control group, with concentrations reaching approximately 47 ng/mL for ALP, 140 ng/mL for OPN, 8 ng/mL for OCN, 0.26 ng/mL for VEGF and 40 ng/mL for AGRIN in the Lv-Slit3 group (Figure 6B).

### Slit3 promotes osteogenesis under inductive conditions

After testing Agrin in the three-microchannel chip model, we tentatively concluded that Agrin functions downstream of Slit3. First, we compared osteogenic capabilities of Slit3 at the early and late stages at the cellular level. The immunofluorescence staining and RT-qPCR results showed that the OIM + Ad-Slit3 group achieved mRNA levels nearly twice those of the control group, whereas interference with Slit3 expression led to a marked reduction in RUNX2 mRNA levels (Figure 7A and B). These results indicated that Slit3 acts as an important positive regulator of the initiation stage of RUNX2-driven osteogenic differentiation. Subsequently, we monitored the mid-to-late osteogenic marker OPN and found that its expression trend was consistent with that of RUNX2 on days 9 and 11 (Figure 7C and D), confirming that the regulatory effect of Slit3 extends through both the early and mid-stages of osteogenic differentiation. We then performed temporal monitoring of the entire osteogenic differentiation process by ALP staining and Alizarin Red staining on days 5, 7, 14 and 21 (Figure 7E). At the early differentiation stage on day 5, no significant difference in ALP activity was observed among the groups. At the mid-to-late stages, on days 7, 14 and 21, the OIM + Ad-Slit3 group exhibited stronger ALP activity and more abundant calcium nodule deposition than the other groups. Notably, interfering with Slit3 expression significantly inhibited the mineralization process. These results demonstrate that Slit3 acts not only at a specific stage but exerts a continuous promoting effect on the initiation, maturation and mineralization of osteogenic differentiation.

### Agrin contributes to Slit3-mediated osteo-angiogenic interaction

To further verify the regulatory hierarchy, we performed Western blot analysis of Agrin and related osteogenic markers. The results showed that the Ad-Slit3 group exhibited higher Agrin expression compared with the control group. In the Ad-Slit3 + si-Agrin group, the expression levels of RUNX2 and OPN were significantly lower than those in the Ad-Slit3 group, confirming that Agrin is indispensable for Slit3-mediated osteogenic differentiation (Figure 8A). To further dissect the functional contribution of Agrin within the Slit3 signaling axis, we evaluated its role in angiogenesis using short-term assays. The scratching assay demonstrated that, compared with the Ad-Slit3 group, the Ad-Slit3 + si-Agrin group exhibited significantly reduced migration, indicating that Agrin participates in Slit3-mediated endothelial cell migration (Figure 8B). However, in the tube formation assay, Agrin knockdown did not significantly impair vascular network assembly, suggesting that Agrin may not be essential for this more complex morphogenetic process (Figure 8B). This functional divergence suggests that Agrin predominantly contributes to cell migration within the Slit3 signaling axis, while its role in the tube formation process is limited.

### Transcriptional activation of Agrin by Slit3-Robo1 through β-catenin-TCF3

Based on the finding that Agrin is a key downstream effector of Slit3, we further investigated its precise regulatory mechanism. Through systematic experimental verification, we delineated the complete Slit3-Robo1-β-catenin-TCF3 signaling pathway and clarified the molecular basis by which Slit3 regulates Agrin transcription. We first screened the potential Slit3 receptors of the Robo family using gene silencing. The results showed that specific silencing of Robo1 significantly inhibited Agrin expression (Figure 8C), whereas silencing other Robo family members had no such effect, indicating that Robo1 is the key receptor mediating Slit3 signal transduction and that Slit3 initiates the downstream signaling cascade through its binding to Robo1. Based on the literature and our preliminary experiments, we hypothesized that β-catenin may play an important role in this pathway. Western blot analysis demonstrated that silencing β-catenin completely blocked Slit3-induced upregulation of Agrin (Figure 8D). Further Western blotting detection confirmed that overexpression of Slit3 promoted the translocation of β-catenin from the cytoplasm to the nucleus, with a significant increase in nuclear β-catenin levels observed at 24 and 48 h (Figure 8E). The TOP/FOP luciferase reporter assay showed that overexpression of Slit3 significantly increased the TOP/FOP ratio, confirming that Slit3 can effectively activate the transcriptional activity of β-catenin (Figure 8F). To elucidate the specific mechanism by which β-catenin acts after nuclear translocation, we performed bioinformatic analysis using the JASPAR database and identified three potential TCF3 binding sites in the Agrin promoter region (−1512 to −1502, −1023 to −1013, −740 to −730). Chromatin immunoprecipitation assays further confirmed that TCF3 antibody was significantly enriched at these regions, demonstrating that TCF3 directly binds to the Agrin promoter and activates its transcription (Figure 8G and H). This finding reveals that β-catenin forms a transcriptional complex with TCF3 that directly binds to the Agrin promoter and activates its transcription.

### Agrin‑mediated osteogenic effect of Slit3 confirmed by Micro‑CT and histology

Based on our previous elucidation of the molecular mechanism of the Slit3–Agrin signaling axis, we further evaluated its biological function in osteoporosis using an OVX mouse model. We first established the classic OVX model of postmenopausal osteoporosis and constructed rAAV9-Slit3 to overexpress Slit3 (Figure 9A). The rAAV9 serotype was chosen for its ability to transduce osteoblast-lineage cells [16]. Immunofluorescence staining and Western blot analysis of femoral tissue revealed that, relative to the sham group, both the immunofluorescence intensity and the protein level of Slit3 were significantly upregulated in the OVX group (Figure 9B). This result not only validated the model but also indicated that endogenous Slit3 may participate in a compensatory repair mechanism during osteoporosis. We then performed a comprehensive assessment at the whole-animal level using micro-computed tomography and histological staining. At week 9, high-resolution micro-CT 3D reconstruction demonstrated that mice in the OVX + rAAV9-GFP group exhibited typical osteoporotic phenotypes, characterized by extensive trabecular bone loss and degeneration of the bone microstructure (Figure 10A). In sharp contrast, the bone architecture of the OVX + rAAV9-Slit3 group resembled that of the sham group, displaying a marked reversal of the osteoporotic phenotype. Importantly, when Agrin expression was simultaneously interfered with by rAAV9-amiR-Agrin, the bone-protective effect of Slit3 was partially attenuated, resulting in moderate bone loss. This observation indicates that Agrin is a key downstream mediator of the osteo-inductive action of Slit3. Quantitative morphometric analysis of bone microarchitecture showed that compared with the OVX + rAAV9-GFP group, the OVX + rAAV9-Slit3 group had significantly higher bone volume fraction (BV/TV), trabecular number (Tb.N), trabecular thickness (Tb.Th) and bone mineral density (BMD), together with significantly lower trabecular separation (Tb.Sp) (Figure 10B). Notably, the OVX + rAAV9-Slit3 + rAAV9-amiR-Agrin group exhibited intermediate values for all microarchitectural parameters between those of the OVX model group and the Slit3-only treatment group, further supporting a pivotal role for Agrin in the Slit3 signaling pathway. Histological analysis by hematoxylin and eosin staining and Masson’s trichrome staining yielded results consistent with the micro-CT data. The OVX + rAAV9-Slit3 group showed a denser trabecular network and abundant deposition of collagen fibers, whereas co-administration of rAAV9-amiR-Agrin substantially diminished this protective effect. These *in vivo* findings confirm that the osteo-inductive effect of Slit3 is mediated through Agrin, thereby corroborating our earlier cellular and molecular data.

**Figure 9:**
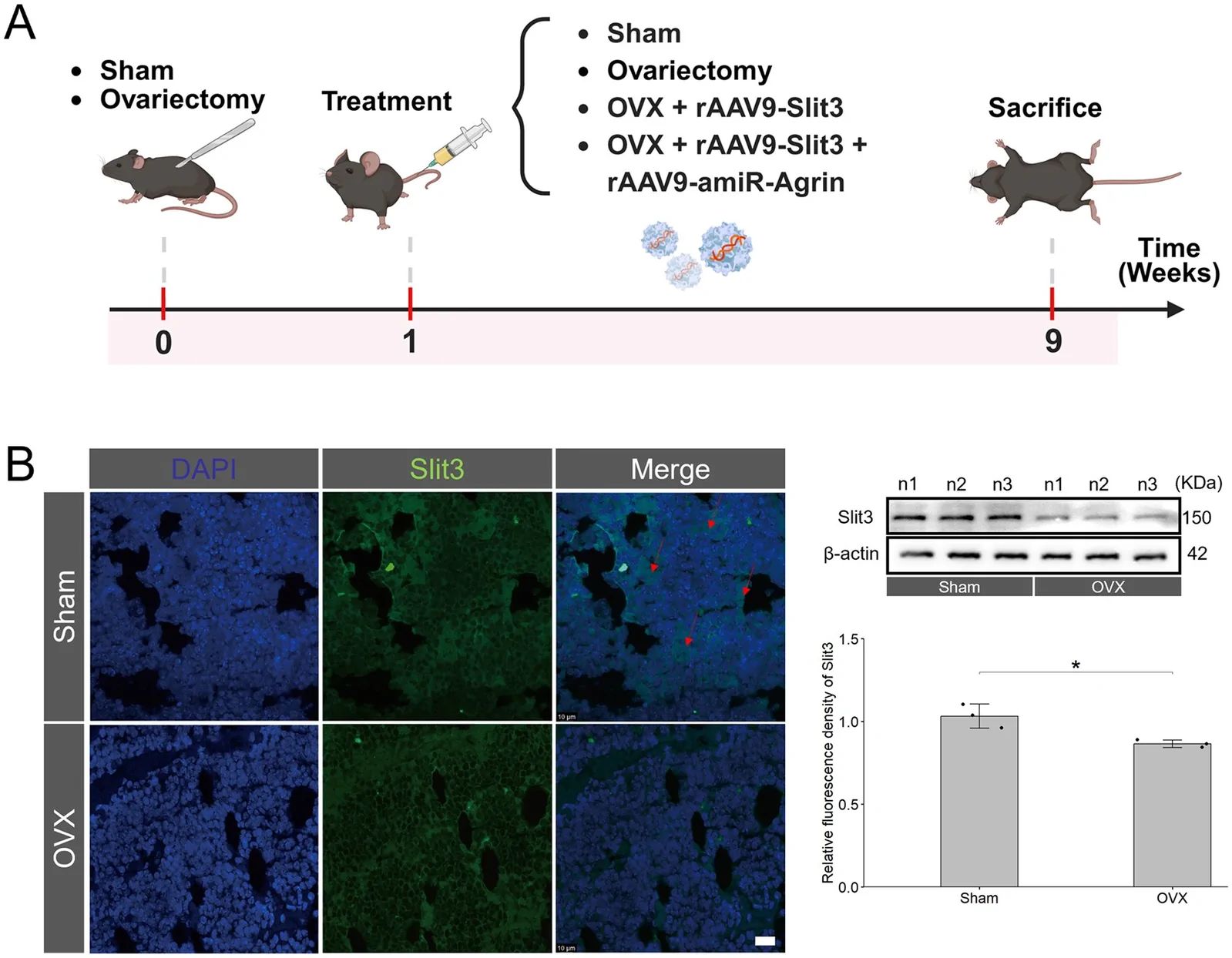
Construction of OVX animal model and verification of Slit3 expression. (**A**) Experimental protocol and timeline for establishing the animal model. (**B**) Immunofluorescence staining and Western blotting detection of Slit3 in femur of the mice. Red arrows indicate Slit3-positive regions. Scale bar = 10 μm. Statistical comparisons were performed by Student’s *t*-test.

**Figure 10:**
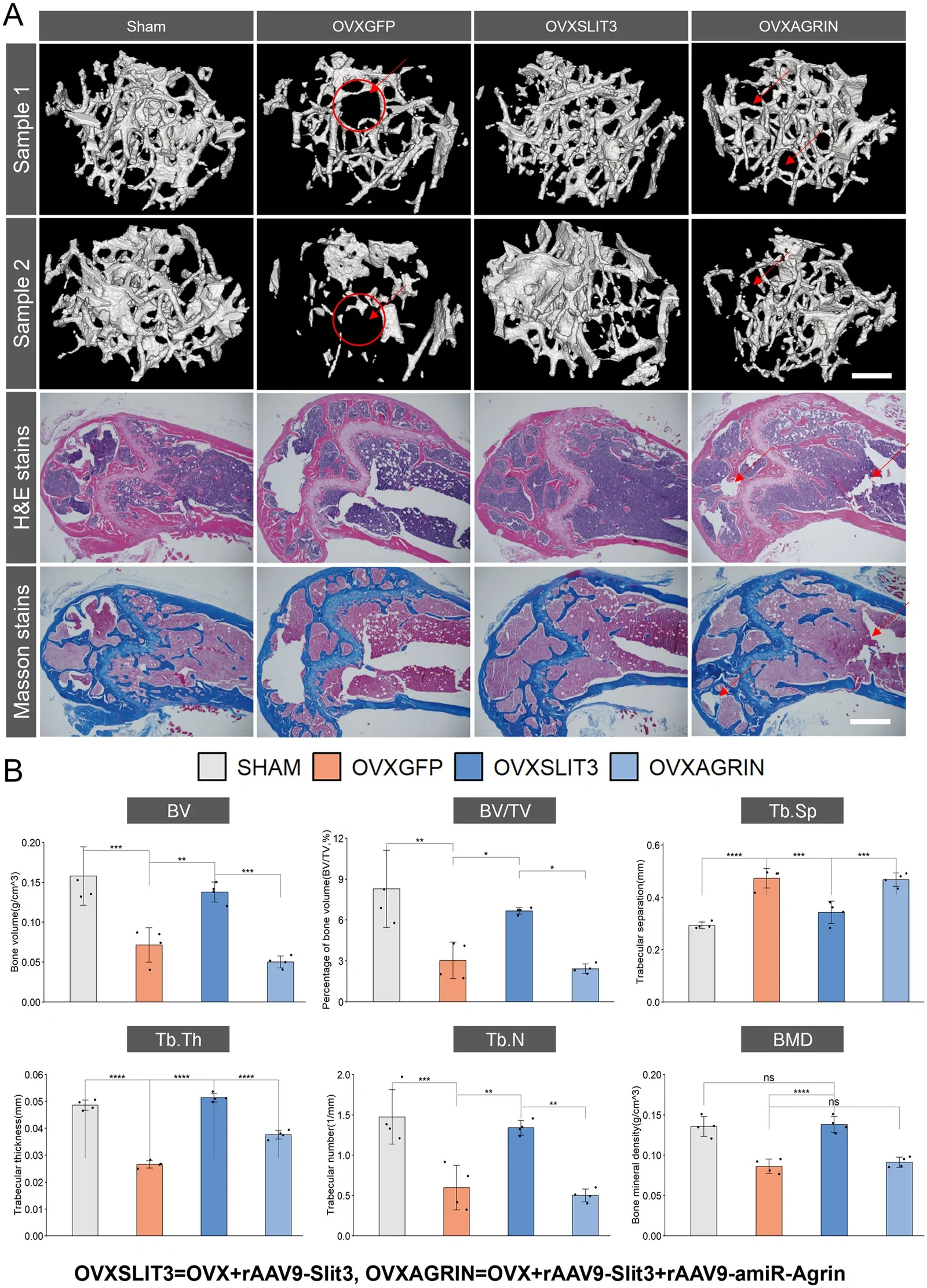
Animal experiments were assessed using micro-computed tomography and histological examination of HE and Masson staining. (**A**) Micro‑CT and histological analysis were performed on the sham operation group, the OVX + rAAV9‑GFP group, the OVX + rAAV9‑Slit3 group and the OVX + rAAV9‑Slit3 + rAAV9‑amiR‑Agrin group. Scale bar = 200 μm. (**B**) Quantitative analysis of the micro‑CT data was conducted, including bone volume (BV), BV/TV, Tb.Sp, Tb.Th, Tb.N and BMD. Statistical comparisons were performed by one-way ANOVA.

## Discussion

This study progressed from 2D culture to a 3D microfluidic chip model, and then to cell-based experiments and an animal model, to systematically dissect the role of the Slit3–Agrin axis in osteo-angiogenic interaction. The *in vitro* model we established not only overcame the limitations of traditional static co-culture, which cannot recapitulate key physiological features such as dynamic interstitial fluid flow and mechanical stress, but more importantly, its three-channel design provided an effective platform for evaluating downstream targets of Slit3. Notably, we optimized the stability of type I collagen hydrogel within the chip and substantially alleviated its spontaneous contraction through a centrifugal compression strategy, enabling the matrix to maintain structural integrity for 21 days. The centrifugal compression was applied uniformly to all experimental conditions before cell seeding. Therefore, any changes from induced compression in matrix physical properties, such as increased stiffness, altered fiber architecture or enhanced ligand density, were identical across the groups.

Furthermore, our findings underscore the complexity of the Slit3 signaling pathway. We initially hypothesized that the osteogenic and angiogenic effects of Slit3 originate predominantly from its downstream secreted protein Agrin. However, the experimental data showed that when Agrin was inhibited, some of the biological effects of Slit3, particularly its pro-angiogenic activity in the tube formation assay, were not completely eliminated. This suggests that, beyond Agrin, additional effector molecules or alternative pathways may operate downstream of Slit3. Multiple independent groups have confirmed the robust pro-angiogenic effect of Slit3, which is consistent with our observations and further supports the view that Slit3 engages diverse downstream signals. Integrating our experimental evidence, we conclude that Agrin is a key downstream mediator of the osteogenic effect of Slit3. Inhibiting Agrin expression indeed attenuates Slit3-driven bone formation, which establishes the necessity of Agrin but also points to the existence of parallel pathways. In summary, Slit3 likely functions as a signaling hub that integrates multiple upstream inputs and precisely orchestrates the interplay between osteogenesis and angiogenesis through several downstream branches, including Agrin.

Another notable finding is that Slit3 activates the β-catenin/TCF3 transcriptional complex through the Robo1 receptor, thereby directly regulating Agrin expression (Supplementary Figure S5). Nevertheless, a key step in this process, how β-catenin converts receptor-proximal signals into nuclear translocation, remains to be elucidated. It is worth noting that Chang *et al*. [17] reported as early as 2012 that Slit2-Robo1 can suppress tumor formation by inhibiting the PI3K/Akt/β-catenin pathway. In contrast, our KEGG enrichment analysis showed that the PI3K/Akt pathway was significantly activated upon Slit3 overexpression, raising the possibility that Slit3 promotes β-catenin activation through positive regulation of the PI3K/Akt pathway. Given the central role of the PI3K/Akt pathway in governing the fate decisions of osteoblasts, chondrocytes and mesenchymal stem cells, this hypothesis is plausible but requires further experimental validation. With respect to the downstream functional network of Agrin, we briefly review the relevant literature to provide a broader context. Agrin, a heparan sulfate proteoglycan, was reported in 2007 by Hausser *et al*. to be highly expressed in chondrocytes and to play a critical role in normal skeletal growth [18]. Mice lacking Agrin die at birth from respiratory failure, underscoring its importance during bone development. That study also suggested that Agrin might be required for Wnt signaling. In 2008, Zhang *et al*. [19] demonstrated that Agrin binds to the LRP4 receptor at the neuromuscular junction, leading to MuSK tyrosine phosphorylation and AChR clustering, highlighting its core function in NMJ formation. In 2015, Eldridge *et al*. [20] showed that Agrin knockdown reduced glycosaminoglycan content and downregulated the cartilage transcription factor Sox9 as well as other cartilage-specific ECM molecules, identifying Agrin as a potential factor in cartilage regeneration. In 2018, the same group reported that Agrin inhibits canonical Wnt signaling through LRP4 and enhances chondrocyte differentiation [21], and in 2020 they further proposed that this Wnt-inhibitory mechanism involves CREB activation by Agrin, which then promotes chondrogenic differentiation [22]. In 2020, Eldridge *et al*. [23] also began to develop Agrin-based hydrogels for cartilage defect repair. In 2021, Souza *et al*. [24] noted that Agrin had been detected in chondrocytes and endosteal osteoblasts but that its function in osteoblast differentiation remained unexplored. They concluded that an Agrin-Wnt-BMP circuit is involved in osteoblast differentiation. Taken together, these studies indicate that Agrin functions not only in neuromuscular junction formation but also in chondrogenic and osteoblast differentiation.

Combining these findings with our own results allows a more complete regulatory network to be constructed. Agrin produced in response to Slit3 may serve as a molecular brake on classical Wnt signaling, shifting cells from a proliferation-oriented Wnt/β-catenin program to a differentiation-oriented CREB-dependent program. This transition has profound implications for bone development. First, it may mark the initiation of the mid-stage of endochondral ossification, because timely termination of Wnt/β-catenin signaling is required for the differentiation of pre-hypertrophic chondrocytes. Second, CREB activation is not only associated with neuronal survival but can also synergistically enhance Runx2 transcriptional activity together with BMP-activated Smad signaling, thereby promoting osteogenic differentiation. Moreover, we detected substantial Agrin expression on day 14, a time point that coincides with the mid-stage of endochondral ossification, and the known regulatory effect of Agrin on Sox9 further supports its role in chondrocyte differentiation. The functional specialization within the LRP receptor family is particularly compelling. Although LRP5/6 serve as co-receptors for classical Wnt signaling, their family member LRP4 functions as the receptor for Agrin and antagonizes Wnt signaling [25]. This division of labor among receptors may provide a precise temporal control mechanism for bone development. On the basis of these converging lines of evidence, we propose that Slit3-regulated Agrin may act as a molecular bridge linking neuromuscular junction formation, cartilage differentiation and osteogenic differentiation by terminating classical Wnt/β-catenin signaling and initiating a CREB-dependent differentiation program, thereby playing a key role in the mid-stage of endochondral ossification. This model not only deepens our understanding of the molecular mechanisms governing bone development but also provides a new conceptual framework for targeting the Slit3–Agrin axis in the treatment of bone diseases. Future studies need to clarify the precise role of PI3K/Akt in Slit3-Robo1 signal transduction and to delineate the specific functions of Agrin in different bone-cell types, in order to fully reveal the complexity of this regulatory network. Furthermore, our findings suggest that Agrin promotes both osteogenesis and angiogenesis in a microfluidic co-culture system. Given the critical role of osteogenic–angiogenic interaction in bone repair and regeneration, Agrin emerges as a candidate molecule for therapeutic intervention in conditions such as nonunion fractures, large bone defects and osteoporosis. However, effective and safe targeting requires careful consideration of several strategies and challenges. First, local delivery of recombinant Agrin protein or its functional domains could be integrated into biomaterial scaffolds or hydrogels to enhance bone healing, an approach that has been successfully explored for other growth factors. Second, small-molecule agonists that activate Agrin’s downstream receptors might offer a more stable and cost-effective alternative, although no such compounds are currently available. Third, gene therapy using viral vectors to overexpress Agrin locally in bone defects is theoretically feasible, but safety concerns regarding off-target effects on neuromuscular junctions must be carefully addressed.

Several limitations should be acknowledged. First, the construction of the hydrogel within the three-microchannel microfluidic chip requires further optimization to better approximate the physiological mechanical properties of bone tissue. Second, in the *in vivo* experiments, tail-vein injection of rAAV9 results in systemic transduction rather than osteoblast-specific targeting. Although our histomorphometry data, including micro-CT analysis, HE and Masson staining, and semi-quantitative assessment of trabecular bone parameters, demonstrated enhanced bone mass and improved bone microarchitecture, which functionally point to increased osteoblast activity, we cannot exclude the potential influence of other transduced cell types due to the systemic nature of rAAV9 delivery. Third, the current chip platform has not yet been employed for drug screening, as our present experimental aim was to simulate a relatively physiological process of bone–vascular interaction. Fourth, the co-culture experiments on the chip did not directly demonstrate functional coupling between the two cell types. Instead, they suggested the existence of paracrine or juxtracrine interactions. Importantly, these limitations do not weaken the principal conclusions but instead highlight important directions for future investigation.

## Conclusion

This study identifies Agrin as a key downstream effector of Slit3 and demonstrates that silencing of Agrin abrogates Slit3-induced bone formation. It also reveals a novel signal transduction pathway, Slit3-Robo1-β-catenin-TCF3-Agrin, in which Slit3 promotes the nuclear translocation of β-catenin. β-Catenin then associates with the transcription factor TCF3 to directly activate Agrin transcription. Furthermore, the Slit3–Agrin axis was validated in an hMSCs-based OoC platform. Agrin was found to inhibit the canonical Wnt/β-catenin signaling pathway through the LRP4 receptor, thereby forming a delicate negative feedback regulatory loop. These findings establish that Agrin is a key, but not necessarily the sole, downstream mediator of the osteogenic effects of Slit3 and provide a rationale for exploring Agrin-based therapeutic strategies for osteoporosis.

## Supplementary Material

rbag192_Supplementary_Data

## Data Availability

The data that support the findings of this study are available from the corresponding author upon reasonable request.
