# Supplementary material for: The role of agrin as a downstream mediator of Slit3 in osteogenic-angiogenic interaction under osteoporosis
Source: Regen Biomater. 2026 Sep 9;13:rbag192. doi: 10.1093/rb/rbag192 (PMC13651701; doi:10.1093/rb/rbag192)
Supplement: Supplementary file 1 [file rbag192_supplementary_data.docx]

**SUPPLEMENTAL INFORMATION**

**The Role of Agrin as a Downstream Mediator of Slit3 in Osteogenic-Angiogenic Interaction Under Osteoporosis**

Xu Ye^#1,2^, Zhenghao Xu^#1^, Gan Zhong^#1^, Wenge He^1^, Yichi Zhang^1,2^, Leilei Qin^1^, Cheng Chen^1^, JianYe Yang^1,6^, Li Chen^1^, Huan Yao^1^, Yingkun Hu^1^, Chengqiang Zhou^1^, Qinyang Zhang^1^, Huimei Liang^1^, Xuan Gong^3^, Denghui Xie^4^, Baicheng He*^5^, Zhong Alan Li*^2^, Ning Hu*^1^

^1^ Department of Orthopedics, The First Affiliated Hospital of Chongqing Medical University, Chongqing, 400016, China;

^2^ Department of Biomedical Engineering, The Chinese University of Hong Kong, NT, Hong Kong SAR, 999077, China;

^3^ Department of Outpatient Care, Chongqing General Hospital, Chongqing, 401147, China;

^4^ Department of Joint Surgery and Sports Medicine, Center for Orthopedic Surgery, The Third Affiliated Hospital of Southern Medical University, Orthopedic Hospital of Guangdong Province, Guangzhou, 510630, China;

^5^ Department of Pharmacology, Chongqing Medical University, Chongqing, 400016, China;

^6^ Orthopedics Department, Chongqing University Fuling Hospital, Chongqing, 408107

\# These authors contribute equally.

*Corresponding Authors: Baicheng He, Zhong Alan Li, Ning Hu

Email: [bche@cqmu.edu.cn](mailto:bche@cqmu.edu.cn), [alanli@cuhk.edu.hk](mailto:alanli@cuhk.edu.hk), [huncqjoint@yeah.net](mailto:huncqjoint@yeah.net)

Figure S1. Flowchart of standard soft photolithography process.

Figure S2. Primers for RT-qPCR.

Figure S3. Schematic diagram of the mutant TCF binding sites in the TOP/Flash and FOP/Flash experiment. Red arrow presents the mutant site.

Figure S4. Tube formation assay on type I collagen. Branch points and tube lengths were quantified by Image J. Statistical comparisons were performed by Student’s t-test. Scale bar=100 μm.

Figure S5. Schematic diagram of the molecular mechanism between Slit3 and Agrin.
